# A seasonal matrix population model for ixodid ticks with complex life histories and limited host availability

**DOI:** 10.1002/ecy.4511

**Published:** 2025-01-15

**Authors:** Yngvild Vindenes, Atle Mysterud

**Affiliations:** ^1^ Centre for Ecological and Evolutionary Synthesis (CEES), Department of Biosciences University of Oslo Oslo Norway; ^2^ Norwegian Institute for Nature Research (NINA) Trondheim Norway

**Keywords:** host–parasite interaction, plasticity, seasonality, stage structure, vector

## Abstract

Many vector‐borne diseases are sensitive to changes in land use and climate; hence, it is important to understand the factors that govern the vector populations. Ixodid ticks, which serve as vectors for multiple diseases, have a slow life cycle compared with many of their hosts. The observable questing population represents only a fraction of the total tick population and may include overlapping cohorts in each stage. The duration of each life stage (larvae, nymph, and adult) is variable and depends on factors such as the seasonal timing of questing, development, and host availability. Mathematical models are therefore essential to mediate how complex life cycle transitions and host interactions underpin the seasonal dynamics of the questing tick population. In this study, we develop a seasonal matrix population model for ixodid ticks feeding on a small and large host. The model has 17 stages representing the main life history stages (eggs, larvae, nymphs, and adults) combined with status of feeding, seasonal timing of feeding, and overwintering. The probability of finding a host depends on tick instar and host type, and density regulation is incorporated through limited host capacity. Using a life history representing *Ixodes ricinus* in Northern Europe as a baseline, we extract seasonal numbers of different parts of the tick population and calculate life history outcomes such as generation time and mean and variance of lifespan and of lifetime reproductive output. These results are compared with an alternative scenario of a southern life history. Secondly, we investigate (1) effects of seasonality in the small host availability on the seasonal numbers of tick stages and (2) effects of varying host availability and utilization of small versus large hosts by larvae and nymphs, on the seasonal numbers of questing ticks. Our results suggest that the small host availability is an important regulating factor through the feeding of larvae. Our model incorporates complex mechanisms underlying the seasonal composition of the tick population. It can be applied to different ixodid tick species and provides a framework for future investigations into intra‐ and interspecific variation in life history and population dynamics.

## INTRODUCTION

Under global change, vector‐borne diseases are becoming a growing threat to human and animal health (Jones et al., 2008; Ogden, 2017). Many of these diseases are transmitted by arthropod vectors, which carry pathogens from reservoir hosts to new hosts, potentially leading to pathogen spillover into human populations (Hill et al., 2005). Arthropod vectors vary in numerous aspects of their biology, including host specificity, degree of association with hosts, and host‐seeking behavior (Ogden & Lindsay, 2016). Understanding the regulation of vector populations is important for predicting disease occurrences and implementing effective risk mitigation strategies. However, this task is challenging due to the diverse and complex life cycles of these vectors. While mosquito‐borne diseases have received much research attention (Reiter, 2001), tick‐borne diseases are not as well studied, despite the fact that ticks represent a distinct group of vectors with unique and varied ecological characteristics (Ogden & Lindsay, 2016). Argasid (soft) ticks, for example, predominantly dwell in close association with their hosts, often within nests, which provides some level of protection against environmental conditions. Conversely, ixodid (hard‐bodied) ticks spend most of their life cycle off‐host, in various stages including diapause, development, and questing (actively seeking hosts), leaving them more exposed to environmental factors (Kahl & Gray, 2023).

In the Northern Hemisphere, pathogens causing Lyme disease, tick‐borne encephalitis (TBE), anaplasmosis, and babesiosis are all transmitted by generalist species of ixodid ticks questing in the environment (Wu‐Chuang et al., 2021). These species include *Ixodes ricinus* in Europe, West Asia, and North Africa; *Ixodes persulcatus* in Asia and Eastern Europe; and *Ixodes scapularis* and *Ixodes pacificus* in North America (Franke et al., 2013). The life cycle of ixodid ticks consists of four main stages: egg, larva, nymph, and adult. To progress to the next stage or reproduce, the larva, nymph, and adult stages require a blood meal from a vertebrate host (Mannelli et al., 2012). Environmental changes can have different impacts on each life stage (Gatewood et al., 2009). Additionally, the synchrony in questing of tick larvae and nymphs can differ depending on local climate conditions (*I. ricinus*; Estrada‐Peña & Estrada‐Sánchez, 2014). Previous models for tick population dynamics have yielded valuable insights on the influence of climate variables, such as temperature and saturation deficit affecting questing and molting (*I. scapularis*: Ogden et al., 2005, *I. ricinus*: Dobson et al., 2011; Li et al., 2016). While the role of seasonality in tick questing and molting is well recognized, less is known about the effects of seasonal changes in the main host species that regulate tick populations.

Several host species, particularly small mammals such as rodents and shrews, exhibit strong seasonality in abundance (Andreassen et al., 2021; Tkadlec & Zejda, 1998). These small mammals, along with ground‐feeding birds, are the main hosts for tick larvae (hereafter termed “small hosts”). Nymphs, on the other hand, feed on a wide range of hosts, including small mammals and birds, while adult female ticks require a blood meal from a larger host (hereafter termed “large hosts”), typically deer, to reproduce (Estrada‐Peña et al., 2016; Hofmeester et al., 2016; Mannelli et al., 2012). The population of larvae is typically an order of magnitude larger than that of nymphs (van Duijvendijk et al., 2015), but the extent to which the nymph population is regulated by the feeding success of questing larvae, and depending on the availability of small hosts, remains uncertain. Most species of voles, mice, and shrews have a high reproductive capacity under favorable conditions and can increase by several orders of magnitude throughout the season (Andreassen et al., 2021). However, the reproductive season of small hosts starts relatively late in northern ecosystems (May/June), resulting in peak density occurring in the fall. Additionally, small mammal hosts are not accessible for tick feeding until they leave their nest or burrow, which may take up to a month. Consequently, the lowest densities of small hosts coincide with the onset of larval questing in late spring. Thus, the peak availability of small mammal hosts may not necessarily align with the peak questing period of larvae or nymphs. Large hosts, such as deer, have lower reproductive capacities and therefore exhibit more stable population densities throughout the year. However, the availability of large hosts to ticks can also vary seasonally due to migration, as these hosts are highly mobile (Qviller et al., 2013). In northern ecosystems, birds display strong seasonality as many species are migratory.

Furthermore, overwintering status and time of feeding affect tick survival, transition, and reproduction, and these factors play a key role in diapause patterns and the duration of the tick life cycle (Gray et al., 2016; Grigoryeva & Shatrov, 2022). For instance, the population of questing nymphs can consist of individuals that have (1) fed and molted earlier in the same year, (2) fed the previous year and overwintered as fed prior to molting, (3) fed and molted the previous year and overwintered as unfed, or a combination of these scenarios (Grigoryeva & Shatrov, 2022; Randolph et al., 2002). Tracking overwintering status is essential for understanding the dynamics because individuals that have overwintered may have different probabilities of survival, questing (if unfed), and molting (if fed) compared with those that hatched/molted/fed in the current year. For unfed ticks, for example, the stored fat reserves from the previous meal will be gradually depleted, resulting in fewer resources for overwintered individuals (Randolph, 2004; Randolph et al., 2002). These ticks may be more inclined to quest under adverse conditions than newly emerged ticks. Additionally, seasonal timing of feeding is important as photoperiod acts as an important cue for diapause (Belozerov et al., 2002; Gray et al., 2016). Ticks that feed before a certain threshold in the summer are more likely to initiate the molting process within the same year, whereas ticks that feed after this threshold are more likely to overwinter as fed and molt the following summer (Gray et al., 2016). Since the seasonal timing of feeding has effects on the future probabilities of molting/reproducing and overwintering, it is essential to incorporate it in a dynamical model.

To gain a comprehensive understanding of disease dynamics involving tick vectors, it is also important that population models capture the seasonal dynamics of their multi‐annual life cycles (Dobson et al., 2011). We consider the aforementioned factors the main components of the life cycle of ixodid ticks, essential for understanding their regulation and critical for investigating the currently uncertain role of seasonal host availability for regulation of tick populations. We develop a seasonal matrix population model for ixodid ticks incorporating detailed underlying mechanisms behind the seasonal composition of tick populations. Our model tracks the main life stages of ticks as well as their status in terms of feeding (fed or unfed), seasonal timing of feeding (spring or fall), and overwintering (current or previous). Density regulation is included through limited space on each available host (Karbowiak et al., 2018; Krasnov et al., 2010), leading to host capacity being filled during the months with the highest questing numbers. First, we use the model to calculate several life history outcomes, comparing a baseline scenario of a life history of *I. ricinus* in Northern Europe with a scenario of a Southern European life history with a different phenology of the small host. Second, we compare scenarios of the baseline model with and without seasonality in small host availability. Finally, we compare scenarios for varying levels of the small and large host, and for different small host utilization by tick nymphs and larvae. In addition to the example analyses presented here, this model provides a general framework for comparing the intra‐ and interspecific life history and seasonal dynamics of *Ixodes* species.

## METHODS

### Life cycle of *I. ricinus*

The structure of the matrix model is defined to fit the life cycles of generalist species of ixodid ticks, mainly *I. ricinus*, *I. persulcatus*, *I. scapularis*, and *I. pacificus* (Gray et al., 2016) that are primary vectors for *Borrelia burgdorferi* sensu lato, the causative agent of Lyme disease, and various other pathogens. Although these tick species share the same main life stages (egg, larvae, nymphs, and adults) and exhibit similar patterns of host selection throughout their ontogeny, they can show large differences in the duration and phenology of each life stage. For instance, *I. ricinus* can overwinter in all stages (Grigoryeva & Shatrov, 2022), whereas the eggs and fed adult stage of *I. persulcatus* cannot overwinter (Gray et al., 2016). Within each species, individual variation in stage transitions is presumably influenced by environmental factors and seasonal cues that affect the likelihood of questing in unfed stages and the molting process of fed stages (Gray et al., 2016; Randolph, 2004).

**FIGURE 1:**
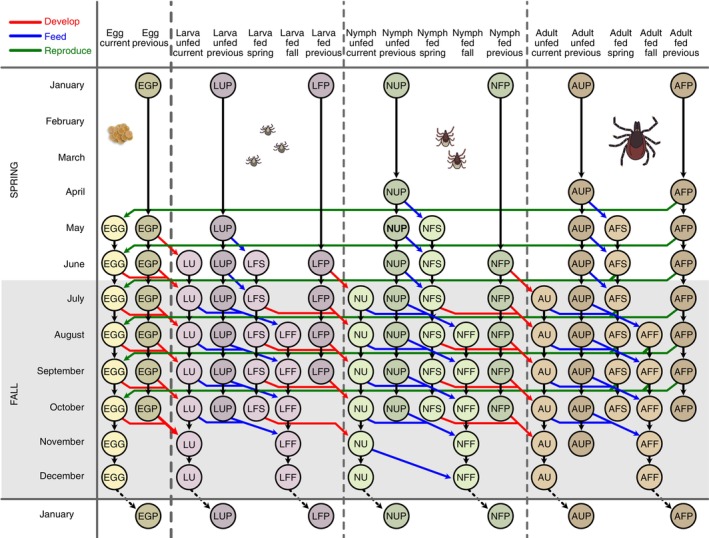
Overview of monthly transitions in the tick model, with possible transitions reflecting a baseline scenario for *Ixodes ricinus* in Northern Europe. Developmental transitions are shown in red and represent hatching for eggs and molting for larvae and nymphs. Feeding transitions are shown in blue. The probability of feeding is density‐dependent in this model, determined by the availability of small and large hosts relative to the number of ticks that quest and find a host. Reproduction is shown in green; females that reproduce die after laying eggs. The model distinguishes between individuals that feed in spring or fall, with a threshold set to July in the baseline scenario. Fall‐fed individuals are more likely to enter diapause to overwinter and molt the next season. Similarly, individuals that emerge from molting in fall are more likely to overwinter as unfed and quest the following year, although some may quest in fall. Overwintered stages (both fed and unfed) are denoted as “previous.” Illustrations by Yngvild Vindenes.

The parameter values in our baseline scenario are chosen to reflect a life cycle of *I. ricinus* in Northern Europe (Figure 1), a species that shows large flexibility in stage transitions (Kahl & Gray, 2023). In Central and Northern Europe, the molting process of fed individuals occurs during summer and fall and takes several weeks to complete depending on temperature (Kahl & Gray, 2023). Ticks that feed in spring and early summer, prior to July, may proceed to molt in the same year and overwinter as unfed in the next stage (behavioral diapause) or potentially quest in the same fall and, if successful, overwinter as fed. However, ticks that feed later in the season typically postpone molting until the following summer and enter developmental diapause after feeding (Grigoryeva & Shatrov, 2022; Randolph, 2004). Unfed individuals, particularly in the nymph and adult stages, can survive for several months before seeking their next blood meal (Grigoryeva & Shatrov, 2022; Randolph, 2004). They can enter diapause during winter or other periods of unfavorable questing conditions, such as heat spells in the summer. Some adult ticks can even survive for two winters without feeding, while unfed larvae and nymphs that have overwintered will die before the second winter unless they find a host (Grigoryeva & Shatrov, 2022). Due to the many potential overlaps of these processes behind transition probabilities between stages, the time required to complete the life cycle varies greatly depending on environmental conditions and location. The lifespan ranges from two (in warmer regions) to over six years (in colder regions), reflecting adaptations to live in varying environmental conditions (Kahl & Gray, 2023). Furthermore, the plasticity of stage durations results in the potential for overlapping generations within the questing population each year (Bregnard et al., 2021).

The questing phenology of the active stages in *I. ricinus* also vary across different geographic regions. In Northern Europe, such as in Scandinavia, the main questing period for nymphs and adults occurs in the spring, while the peak activity of larvae is in summer (Kahl & Gray, 2023). Conversely, in Southern Europe, the main questing period for nymphs occurs much earlier during winter and early spring, with minimal overlap with the larvae (Dantas‐Torres & Otranto, 2013). In Northern and Central Europe, a bimodal pattern is often observed in the questing activity of nymphs and adults, with a spring peak followed by a smaller peak in the fall (Wongnak et al., 2022). This pattern may arise from different mechanisms, or combination of mechanisms. For example, poor questing conditions during the summer may increase the likelihood of behavioral diapause in the unfed ticks. Alternatively, the different peaks may reflect the emergence of different cohorts of ticks, with a spring peak arising from overwintered unfed nymphs and adults, and a fall peak consisting of ticks that fed the previous year and molted in the current year to emerge in late summer. Furthermore, the fall peak could also include individuals that fed early in the same year and completed the molting process in time to quest in the fall (Bregnard et al., 2021).

Following a blood meal, the ticks can either initiate the molting process to the next stage or enter developmental diapause, a period of developmental arrest lasting several months, helping them to survive winter (Gray et al., 2016; Grigoryeva & Shatrov, 2022).

### Model description

In this section, we describe the model and its main components, as well as our choices for parameter values in the baseline scenario for *I. ricinus* ticks in Northern Europe (Figure 1). Importantly, the model can be adjusted to fit the wide range of variation in local tick life histories and host availability by modifying parameters. A description of key model parameters can be found in Table 1, and additional details are given in Appendix S1. The analyses were done with R version 4.4.1 (R Core Team, 2024), and the R Markdown file with all R code to reproduce Appendix S1 with all main figures is published on figshare (Vindenes & Mysterud, 2024).

**TABLE 1:** Overview of main parameters in the model and baseline values referring to a population of *Ixodes ricinus* in Northern Europe.

Parameter	Description	Baseline value(s)	References
$a_{\mathrm{ji}}$	Survival probability stage j, month i	Appendix S1: Figure S5	Daniel et al. (1976), Gray (1981), Grigoryeva and Shatrov (2022), Randolph (2004)
$q_{\mathrm{ji}}$	Questing probability in stage j, month i	Appendix S1: Figure S5	Daniel et al. (1976), Gray (1981), Grigoryeva and Shatrov (2022), Randolph (2004)
$m_{\mathrm{ji}}$	Molting probability in stage j, month i	Appendix S1: Figure S5	Daniel et al. (1976), Gray (1981), Grigoryeva and Shatrov (2022) Randolph (2004)
$h_{\mathrm{ji}}$	Hatching probability in stage j, month i	Appendix S1: Figure S5	Daniel et al. (1976), Gray (1981), Grigoryeva and Shatrov (2022), Randolph (2004)
$r_{\mathrm{ji}}$	Reproduction probability in stage j, month i	Appendix S1: Figure S5	Daniel et al. (1976), Gray (1981), Grigoryeva and Shatrov (2022), Randolph (2004)
b	Fecundity of ticks, eggs per reproducing female	1500	Gray (1981), Grigoryeva and Shatrov (2022)
$S_{i}$	Small host availability in month i	Appendix S1: Figure S2	Crespin et al. (2002), Nyholm and Meurling (1979)
$L_{i}$	Large host availability in month i	500	
$p_{j}$	Small host utilization in tick instar j (larva, nymph, adult)	$p_{L}=0.9$ $p_{N}=0.5$ $p_{A}=0$	Randolph (2004), Tälleklint and Jaenson (1994)
${\mathrm{MS}}_{j}$	Maximum probability of finding a small host, tick instar j (larva, nymph)	${\mathrm{MS}}_{L}=0.7$ ${\mathrm{MS}}_{N}=0.9$	Randolph (2004), Tälleklint and Jaenson (1994)
${\mathrm{ML}}_{j}$	Maximum probability of finding a large host, tick instar j (larva, nymph, adult)	${\mathrm{MS}}_{L}=0.3$ ${\mathrm{MS}}_{N}=0.6$ ${\mathrm{MS}}_{A}=0.9$	Randolph (2004), Tälleklint and Jaenson (1994)
C	Capacity (feeding spots) per small host	500	Lindsø et al. (2023), Tälleklint and Jaenson (1994)
$C_{j}$	Capacity (feeding spots) per large host for tick instar j	${\mathrm{MS}}_{L}=5000$ ${\mathrm{MS}}_{N}=5000$ ${\mathrm{MS}}_{A}=5000$	Mysterud et al. (2014), Tälleklint and Jaenson (1994)

*Note*: References refer to references with information used to set parameter values. Tick stage *j* refers to the relevant stage out of the 17 stages described in Figure 1. Transitions to “previous” stages occur between December and January.

#### Stage structure and population dynamics

The tick model is female based with a monthly timescale. The tick population is classified into 17 distinct stages (Figure 1; Appendix S1: Table S1), reflecting not only the main life stage (egg, larvae, nymph, or adult) but also the feeding state (unfed or fed), seasonal timing of feeding (spring or fall), and overwintering state (current or previous). Keeping track of overwintering status, for example, is important in the following summer and fall, as overwintered individuals will have a different phenology of questing and development, and unfed ones will eventually have a reduced survival as their fat resources are used (Randolph, 2004). Similarly, spring‐fed individuals are more likely to develop and emerge in fall of the same year, while fall‐fed individuals are more likely to remain in the fed stage over winter and develop and emerge next summer (Gray et al., 2016). The model uses a post‐reproductive census, where individuals in each stage are counted at the start of each month. During the month, individuals may survive and make a transition to a different stage or reproduce, depending on the month and their current stage. For instance, eggs may hatch, unfed individuals may quest and feed, fed larvae and nymphs may molt, and fed adults may reproduce. Reproducing adults in the model lay their eggs at the start of the next month and then die right before the subsequent census. We assume that adults feeding in the current month cannot reproduce until at least one month has passed since they first entered the fed stage. This effectively ensures a minimum two‐month interval between feeding and egg‐laying. Additionally, the feeding process incorporates density dependence through the availability and capacity of small and large hosts, described in the next subsection.

Denoting the stage‐specific tick population vector at time step t and month i $\left(i=1,2,\ldots ,12\right)$ as $n_{i,t}$, the population size in the next month is given by $n_{i+1,t+1}=A_{i,t}\left(S_{i},L_{i},n_{i,t}\right),$ where $A_{i,t}\left(S_{i},L_{i},n_{i,t}\right)$ is the projection matrix for month i at time t. This matrix depends on the availability of small hosts $S_{i}$, the availability of large hosts $L_{i}$, and the current density of ticks $n_{i,t}$, which together determine the transition probabilities from unfed to fed stages (described in the next subsection). The monthly projection matrix can further be decomposed as $A_{i,t}\left(S_{i},L_{i},n_{i,t}\right)=U_{i,t}\left(S_{i},L_{i},n_{i,t}\right)+F_{i}=T_{i,t}\left(S_{i},L_{i},n_{i,t}\right)\mathrm{diag}\left(a_{i}\right)+F_{i}$, where $F_{i}$ is a density independent fertility matrix for month i, describing reproduction from the three adult fed stages, $a_{i}$ is the vector of stage‐specific background survival probabilities, and $T_{i,t}\left(S_{i},L_{i},n_{i,t}\right)$ is a density dependent transition matrix for month i. The matrix $U_{i,t}\left(S_{i},L_{i},n_{i,t}\right)=T_{i,t}\left(S_{i},L_{i},n_{i,t}\right)\mathrm{diag}\left(a_{i}\right)$ thus represents the combined survival and transition between stages.

The monthly timescale gives a coarse time resolution compared to the daily timescale used in some other models (e.g., Dobson et al., 2011; Li et al., 2016) but has also been used by other studies (e.g., Dobson & Auld, 2016). A monthly scale is sufficient to describe main seasonal patterns given the goals of this study; to calculate life history outcomes and to evaluate effects of host seasonality, host use and availability on seasonal numbers of questing ticks. The model probabilities of survival and transition represent averages within each stage, allowing us to calculate an annual projection matrix when the stable seasonal cycle is reached, using any month as the annual census time, as described below. This enables calculation of a range of life history and demographic outputs such as the stable stage structure and reproductive values, generation time, mean and variance of (remaining) lifespan, and the mean and variance of (remaining) lifetime reproduction, using matrix population model methods (Caswell, 2001, 2009; Hernández et al., 2024). Other models such as the model by Dobson et al. (2011) incorporate some transitions (such as molting) as fixed individual‐based processes simulated outside of the projection matrix. This allows more detailed physiological processes to be included but precludes the calculation of life history outcomes based on matrix model methods. Our model trades off some individual‐level detail for analytical tractability.

#### Host availability and feeding of ticks

**FIGURE 2:**
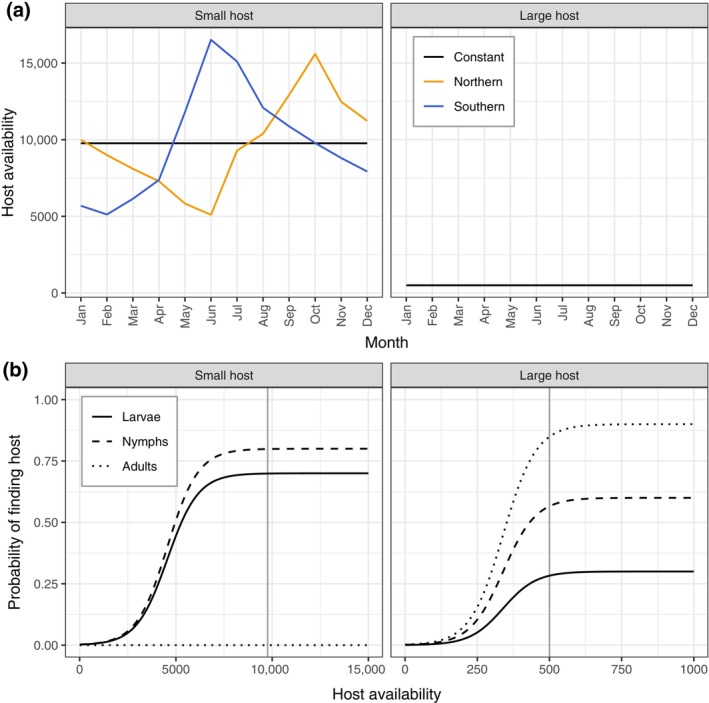
Properties of host availability and feeding used in the analyses. (a) Seasonal availability of small and large host availability used in the different scenarios. The small host availability represents adult individuals of a small mammal. (b) The stage‐dependent probability of ticks finding a small host and large host used in the analyses. Vertical lines represent the mean host availability across scenarios.

Hosts to *I. ricinus* ticks can be functionally divided into (smaller) hosts that only feed larvae and nymphs, and the larger hosts that are required for reproduction by the adult ticks, that also feed nymphs and to a less extent larvae. In the scenarios analyzed in this study, we consider that the small host availability $S_{i}$ shows seasonal variation, while the large hosts availability $L_{i}$ remains constant throughout the year (Figure 2). The transition of larvae, nymphs, and adults from unfed to fed stages in a given month i depends on three main processes: (1) questing, (2) locating a small or large host given questing, and (3) available space to feed given that a host is found. The probability of questing in month i is denoted as $q_{\mathrm{ji}}$, where the stage j represents one of the unfed stages (LU, LUP, NU, NUP, AU, and AUP). The small host utilization $p_{j}$ denotes the average proportion of the questing population within each stage that “search” for small hosts, while the remaining proportion seeks large hosts ($p_{L}=0.9$, $p_{L}=0.5$, $p_{A}=0$ for larvae, nymphs, and adults, respectively). Importantly, this host utilization parameter does not imply that individual ticks actively search for a specific host type. Instead, it reflects the average outcomes of stage‐specific questing behaviors, such as vertical positioning within the vegetation. For instance, larvae tend to quest at lower heights, where they are more likely to encounter small hosts (Dantas‐Torres & Otranto, 2013; Randolph, 2004). The stage‐specific probability of finding a host of a given type is modeled as a logistic function of host availability, with the maximum probability depending on stage and host type (Figure 2b). The probability of a larvae finding a small host is denoted as $f_{L}\left(S_{i}\right)$, the probability of a larva finding a large host is denoted as $f_{L}\left(L_{i}\right)$, and similar notation is used for the other tick stages. We assume that larvae have a lower probability of finding a host or reaching a suitable spot on a host than nymphs and adults, due to their limited mobility.

Whether all ticks that find a host in a given month can feed depends on the host capacity relative to the number of ticks of each instar. We define the following processes for tick feeding in the model: The total number of unfed larvae that find a small host in month i is given by $n_{L}^{*}\left(S_{i}\right)=\left(n_{{\mathrm{LU}}_{i}}q_{{\mathrm{LU}}_{i}}+n_{{\mathrm{LUP}}_{i}}q_{{\mathrm{LUP}}_{i}}\right)p_{L}f_{L}\left(S_{i}\right)$, while the number of larvae finding a large host is $n_{L}^{*}\left(L_{i}\right)=\left(n_{{\mathrm{LU}}_{i}}q_{{\mathrm{LU}}_{i}}+n_{{\mathrm{LUP}}_{i}}q_{{\mathrm{LUP}}_{i}}\right)\left(1-p_{L}\right)f_{L}\left(L_{i}\right)$. Similar expressions apply to the number of nymphs and adults. We assume that each small host has a capacity of C “feeding spots,” where nymphs are assumed to require three feeding spots each and larvae one spot each. Note that the host capacity C is a model parameter that may reflect not only physical space limitation on the host but also behavioral limitation such as grooming or movement away from areas of high tick density, as well as host immune responses (Keesing et al., 2009). The total available spots on small hosts are distributed among the nymphs and larvae that find a small host. If the total number of requested spots by nymphs and larvae is below the total number of available spots (if $n_{L}^{*}\left(S_{i}\right)+3n_{N}^{*}\left(S_{i}\right)\leq CS_{i}$), all ticks that find a small host will feed. If not, the number of larvae that can feed is given by $n_{L}^{**}\left(S_{i}\right)=\left[CS_{i}n_{L}^{*}\left(S_{i}\right)\right]/\left[n_{L}^{*}\left(S_{i}\right)+3n_{N}^{*}\left(S_{i}\right)\right]$, while the number of nymphs that can feed is $n_{N}^{**}\left(S_{i}\right)=\left[CS_{i}n_{L}^{*}\left(S_{i}\right)\right]/\left[n_{L}^{*}\left(S_{i}\right)+3n_{N}^{*}\left(S_{i}\right)\right]$. In our analyses, we assume C=500, meaning that each small host can on average feed 500 larvae or 167 nymphs per month, or a combination of both in between. There is not much empirical information available to support this number; however, it corresponds approximately to reported numbers of ticks on mice and voles scaled up to one month (Lindsø et al., 2023), assuming larvae take 2–4 days per meal and nymphs 3–5 days (Kahl & Gray, 2023). As the population of questing larvae is typically much higher than that of questing nymphs, during months of high competition, larvae will secure most of the available feeding spots on small hosts. For large hosts, the different tick stages are assumed to use different parts of the host (Mysterud et al., 2014) so that ticks only compete within stage. Each large host has a capacity $C_{L}$ for larvae, $C_{N}$ for nymphs, and $C_{A}$ for adults (all set to 5000 in our analyses, assuming that if a tick finds a large host it will be able to feed). Together, the host availability (number of individuals) and capacity (number of ticks that can feed per host) determine how many ticks can feed in a given month and thereby also how many ticks can complete the life cycle. While it is difficult to find empirical estimates for these parameters, it is reasonable to assume that the population of large hosts (e.g., deer) for a given area is generally much smaller than the population of small hosts (e.g., mice, voles, and shrews). If thus, the potential limitation of the tick population through large hosts would more likely be mediated by host numbers and the ability of ticks to locate them than capacity per host, while small hosts may more likely limit the ticks through a combination of both numbers and capacity per host. If the host availability and capacity values are set so low in the model that fewer than one female tick per the average 1500 eggs that are laid can complete the life cycle (1 in 750), the tick population will not be viable.

#### Baseline northern scenario

The baseline parameter values for the model (Appendix S1: Figure S5) are chosen to represent a life history of *I. ricinus* in Northern Europe (Figure 1), characterized by relatively high survival rates due to a low risk of desiccation, but slow average development rates due to low mean temperature (Estrada‐Peña & Estrada‐Sánchez, 2014). The peak questing is set to occur in May for overwintered nymphs, June for adults, and July for larvae (Walker, 2001), with smaller peaks in the fall representing questing from newly emerged individuals (Appendix S1: Figure S5). Nymphs and adults are assumed to have slightly longer questing periods each year than larvae. Late summer is assumed to be the peak season for molting and egg‐laying by adult fed females (Appendix S1: Figure S5). We assume each reproducing female lays 1500 eggs, in line with reported average values for *I. ricinus* (Gray, 1981; Grigoryeva & Shatrov, 2022). The chosen baseline values for survival are based on life history information from Grigoryeva and Shatrov (2022) and Randolph (2004), and references within these, as described Appendix S1: Section S2.2. Our values are within range reported by empirical observation studies (Appendix S1: Table S2; Daniel et al., 1976; Gray, 1981; Grigoryeva & Shatrov, 2022; Randolph, 2004). We assume the survival is slightly higher in the fed than in unfed stages, as questing and feeding is associated with a higher risk of mortality; however, this increased risk is also implicitly included in the probabilities of questing in our model. Our survival values are generally a bit lower than estimates from observational studies (Appendix S1: Table S2), which likely overestimates survival compared with natural populations (Randolph, 2004). We assume that overwintered stages have a progressively lower survival rate throughout the year as their stored fat resources are depleted. Survival in these stages eventually reaches zero in the model, meaning overwintering individuals will die during the following year unless they successfully feed or reproduce in the case of adult fed females. Grigoryeva and Shatrov (2022) also observed that up to 9% of adult unfed females could overwinter twice; this possibility is not included in our model.

Photoperiod‐induced diapause is accounted for through the separation of spring‐ and fall‐fed ticks, which have different probabilities of molting. In January, fed ticks that have not molted will enter the “fed previous” stage. July is set as the threshold for spring‐ and fall‐feeding, corresponding to Northern Europe (Gray et al., 2016). Ticks that feed before July can molt later in the same year, then overwinter as unfed and quest next year. Larvae and nymphs that feed from July onward are assumed to overwinter as fed and complete the molting process in the following summer. These individuals may then quest in fall that year or overwinter as unfed, generating a one‐year delay in the life cycle (Grigoryeva & Shatrov, 2022). Adults that feed in spring are assumed to lay their eggs the same year, along with a few adults that feed in fall. But in the baseline scenario, we assume most of the fall‐fed adults will overwinter and lay their eggs next year. Our choices regarding duration and peak periods of hatching, questing, and molting in different stages are based on the information provided by Grigoryeva and Shatrov (2022), Randolph (2004), and Kahl and Gray (2023); however, exact empirical estimates of monthly probabilities are not available for these processes. We therefore tuned the magnitude of these parameters in initial model development to obtain a baseline model with (1) generation time of 3–4 years, (2) a viable tick population, and (3) a resulting population size of questing nymphs being approximately one order of magnitude lower than that of larvae (van Duijvendijk et al., 2015).

For the southern scenario, we adjust the phenology of the tick processes (Appendix S1: Figures S36 and S37) to fit approximately with the information provided by Dantas‐Torres and Otranto (2013) for a population from southern Italy and by Estrada‐Peña et al. (2004) for a population from north‐central Spain. Survival in this scenario is assumed to be 3%–8% lower than in the northern population due to an increased risk of desiccation (Estrada‐Peña & Estrada‐Sánchez, 2014). The transitions through the life cycle are faster than in the north, so we assume that eggs are more likely to hatch in the same year as they are laid. Furthermore, all larvae are assumed to quest in the same year as they are hatched (no overwintering of unfed larvae). The nymphs have two questing peaks, one in winter and one in fall, while adults have one questing peak in winter/early spring and larvae in late summer (Appendix S1: Figure S37).

### Model outputs

For each scenario and parameter combination, we project the monthly population growth for several years, tracking each stage and storing each monthly transition matrix, until well after a stable seasonal cycle is reached (visually assessed). The monthly projection matrices are then the same each year, and the population will show only seasonal fluctuations (no interannual fluctuations). While it is theoretically possible to obtain multi‐annual cycles with this model, most parameter combinations including all scenarios considered here will lead to only one stable seasonal cycle.

We extract the seasonal number of questing, feeding, fed, and emerging ticks from each of the main active stages (larvae, nymphs, and adults). For the seasonal questing numbers, we separate between different generations (current or previous). For the monthly numbers of successfully feeding individuals, we separate between host type (small or large), and we also calculate the monthly proportion of host capacity that is used, which indicates months of density regulation (Appendix S1: Sections S3.1, S3.3, S3.4). Regarding the fed population, we separate individuals that fed in spring, fall, and the previous year (Figure 1). For newly emerging (molted) individuals, we track whether they come from overwintered individuals that fed the previous year or from individuals that fed earlier in the same year.

For the equilibrium populations, we calculate the annual projection matrix and its main components and use these to calculate the long‐term annual growth rate λ, stable stage structure u, and reproductive values v. In addition, we calculate the mean and SD of lifespan and stage‐specific (remaining) lifespan, the mean and SD of lifetime reproductive output and stage‐specific (remaining) lifetime reproductive output, and the generation time, using methods described by Hernández et al. (2024). These life history outputs provide information not only for the mean trajectory of individuals through the stages but also for the amount of variation in these trajectories. We verify that the annual growth rate λ and the net reproductive rate $R_{0}$ (mean lifetime reproductive output) are equal to 1 (due to density regulation). We do the same calculation for 12 different annual projection matrices, one for each month as the annual census month. For instance, the annual projection matrix from August to August is given by $A_{\text{August}-\text{to}-\text{August}}=A_{7}A_{6}A_{5}A_{4}A_{3}A_{2}A_{1}A_{12}A_{11}A_{10}A_{9}A_{8}$, where $A_{i}$ is the extracted projection matrix for month i. The stable stage structure u and the reproductive values v are calculated as the right and left eigenvectors of the projection matrix $A_{\text{August}-\text{to}-\text{August}}$ associated with the dominant eigenvalue (Caswell, 2001). To extract the annual survival/transition matrix $U_{\text{August}-\text{to}-\text{August}}$, we use the same approach as for the annual projection matrix (multiplying monthly survival/transition matrices extracted at equilibrium). The annual fertility matrix is $F_{\text{August}-\text{to}-\text{August}}$ = $A_{\text{August}-\text{to}-\text{August}}-U_{\text{August}-\text{to}-\text{August}}$ (Caswell, 2009). Generation time is calculated as the mean age (in years) of reproducing females for the population at the stable stage structure (Bienvenu & Legendre, 2015), $G=\frac{\lambda }{\mathrm{vFu}}=\frac{1}{\mathrm{vFu}}$.

## RESULTS

### Baseline northern scenario

**FIGURE 3:**
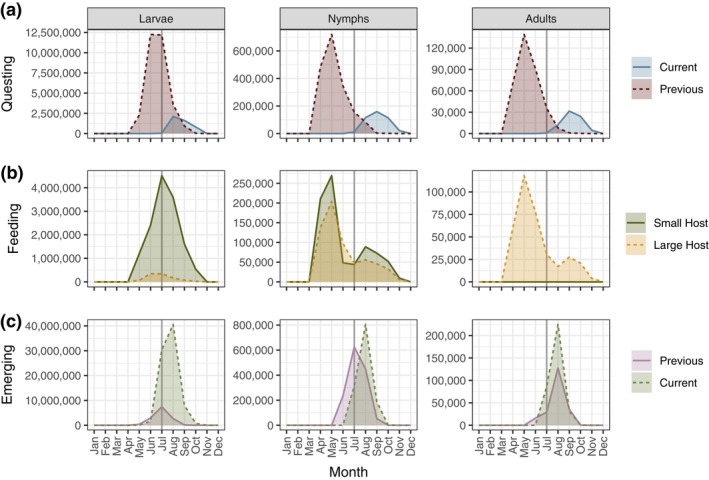
Model outputs at the equilibrium stable state in the baseline northern scenario with a seasonal small host (solid lines) and the corresponding outputs for the same model with a constant small host (dashed lines). (a) Questing population of larvae, nymphs, and adults from individuals that emerged as unfed in the current year and from individuals that emerged in the previous year and overwintered as unfed (previous). (b) Numbers of larvae, nymphs, and adults that feed on each host type each month. (c) Numbers of emerging (unfed) larvae, nymphs, and adults each month, divided into those that fed earlier in the same year (current) and those that fed previous year and overwintered as fed (previous).

The model for the baseline northern scenario has a generation time of 3.72 years at equilibrium (Appendix S1: Table S3). During the peak questing period, the number of questing larvae is an order of magnitude higher than the number of nymphs, which is in turn an order of magnitude higher than the number of adults (Figure 3a). The peak month of the questing population mostly corresponds to the peak month of questing probability (Appendix S1: Figure S5), but there is a slight difference for larvae as their peak questing occurs in June, while the peak questing probability is in July. Most questing ticks are individuals that hatched or molted in the previous year, but there is also a smaller fall peak of questing individuals from the current year. Most of the larval feeding occurs in July and August (Figure 3a), after the peak questing period. Since the small host population is larger in fall than in spring (Figure 2a), more questing larvae will successfully feed in fall. In June and July, when competition for small hosts is highest (Appendix S1: Figures S10–S12), the probability of finding a small host is lower (Figure 2), resulting in a smaller proportion of questing larvae feeding in these months. July has the highest number of feeding larvae because a large number are questing relative to the available number of small hosts. In most months, the number of nymphs feeding on small hosts is higher than the number feeding on large hosts, except in June and July when competition with larvae is high. In the other months, all questing ticks that find a host will successfully feed. The adult ticks only feed on large hosts, and their peak feeding period corresponds to the peak in questing (Figure 2b). The peaks for larvae and nymphs feeding on large hosts also align with the peak in questing, as there is no competition for these hosts in the baseline scenario. The stable population structure at equilibrium is skewed toward the egg and unfed larvae (Appendix S1: Figure S8), while the reproductive value increases with each life stage and is highest for adults (Appendix S1: Figure S9). Furthermore, adults that feed in fall have a higher reproductive value than adults that feed in spring (Appendix S1: Figure S9).

The population of fed individuals in the model also varies throughout the year (Appendix S1: Figure S13), increasing as newly fed individuals enter and decreasing due to mortality and transitions out of fed stages. The spring‐fed population reaches its peak during the summer, and these individuals undergo molting (or reproduction) in the summer or fall of the same year. All fall‐fed larvae, nymphs, and most fall‐fed adults in the model enter diapause and complete their molting (or reproduction) in the following year. Figure 3c shows the number of newly emerging (unfed) larvae, nymphs, and adults each month, along with whether they originate from overwintered eggs or previously fed individuals (“Previous”), or from eggs laid and individuals fed in the current year (“Current”). Most of the larvae emerge from eggs laid in the current year, while approximately half of the nymphs emerge from larvae that were fed earlier in the same year, and about half of the adults from nymphs that were fed earlier in the same year.

The life history analyses reflect the high flexibility of the *I. ricinus* life history (Appendix S1: Figures S19–S22). The mean remaining lifespan is generally low in all stages (Appendix S1: Figure S19), reflecting the high mortality in a life history where most individuals never survive to reproduce. For the fed nymphs and the adult stages, however, reproduction is closer in time, so the values are high enough that the likelihood the individual will make it to reproduce is much higher. The SD of remaining lifespan is high throughout most larval and nymphal stages (Appendix S1: Figure S20). As reproduction is fatal in this species, both mean and variance of lifespan are lower in adult stages. The expected lifetime‐reproductive output increases with each instar and is higher the closer the stage is to reproduction. While the overall expected lifetime reproductive output of an average offspring is always 1, there is much variation in this parameter over stage (Appendix S1: Figure S21).

### Varying host levels and host type utilization

**FIGURE 4:**
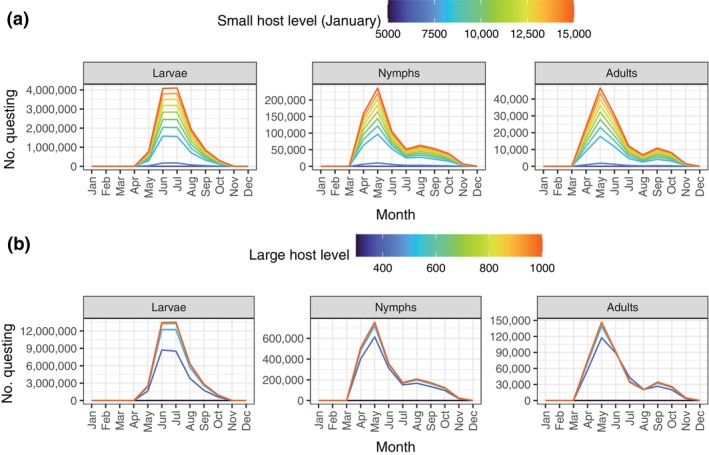
Effects of varying host availability on the equilibrium questing population in the baseline northern scenario. (a) Effects of varying small host level (referring to the initial January size). The large host availability is 500 as in the baseline scenario. (b) Effects of varying the large host level, which is constant across seasons. The small host availability is seasonal as shown in Figure 2.

When varying the host availability, the modeled tick population is more responsive to changes in the small host availability than in the large host availability (Figure 4). Some large hosts are required for reproduction since adult ticks only feed on large hosts. However, increasing the availability of large hosts beyond a certain level has no impact on the tick population size in the model (Figure 4). This suggests that at least under the assumptions of the baseline northern scenario, the tick population is primarily regulated by small host availability controlling the larvae and nymphs.

**FIGURE 5:**
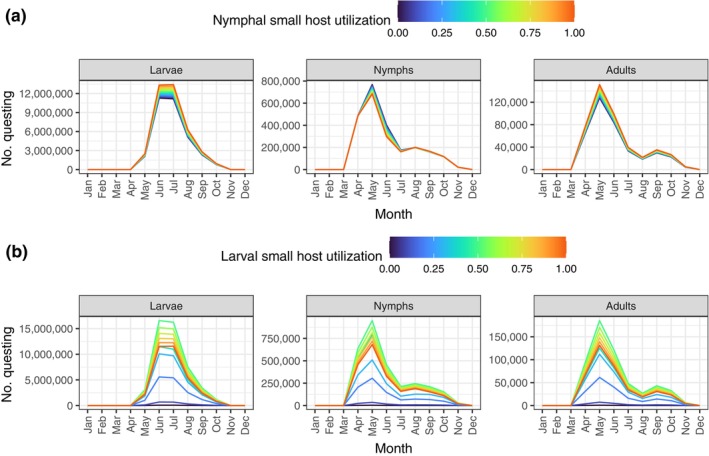
Effects of variation in small host utilization of nymphs and larvae on the equilibrium questing population in the baseline northern scenario. Adults feed only on large hosts. (a) Effects of varying nymph small host utilization. The larval small host utilization is here 0.9. (b) Effects of varying larval small host utilization. The nymphal small host utilization is here 0.5. Results corresponding to larval small host utilization lower than about 0.4 lead to declining, nonviable populations (no equilibrium reached, results shown are after 1000 months).

The tick population is also more sensitive to the small host utilization by larvae than nymphs (Figure 5). If the small host utilization by larvae is very low (below approximately 0.5), the tick population is not viable under the baseline scenario. As long as the tick population is viable, an intermediate value of the small host utilization by larvae leads to the largest tick population size (Figure 5), as it allows for an optimal distribution of questing larvae among small and large hosts to maximize the number that can feed. Additionally, the competition with nymphs for small hosts is reduced during the critical months of June and July. On the other hand, changing the small host utilization by nymphs has a smaller effect (Figure 5). Increasing nymphal utilization of small hosts leads to a higher number of questing larvae and adults, but slightly fewer questing nymphs. As more nymphs feed on small hosts, the competition with larvae will increase in this model during the months of high questing relative to host availability (given the baseline larval small host utilization of 0.9), and since there are more questing larvae overall, they are more likely to obtain a larger share of the available spots on small hosts.

### Constant small host

Comparing the baseline northern model with a model with constant small host availability through the year (Appendix S1: Figure S25), the main effect is an increased tick population due to more larvae and nymphs being able to feed during the months of high questing activity. In addition, the small host capacity is filled for a longer time period (June, July, and August), leading to increased competition between larvae and nymphs during these months. The generation time is only slightly reduced (to 3.6 years compared with 3.7 for the scenario with a seasonal small host), and other life history outcomes are also similar (Appendix S1: Table S4).

### Southern scenario

The results for the southern scenario are presented in Appendix S1: Section S3.4. In this scenario, the mean generation time is 2.2 years, much shorter than the 3.7 years in the northern scenario. The mean and SD of lifespan are also much lower than in the northern scenario, as is the SD of lifetime reproductive output. Furthermore, the peak availability of small hosts occurs earlier in the year (Figure 2a) in comparison with the northern scenario, enabling more larvae to find a host and feed during their peak questing season. Since nymphs and larvae quest at different times of the year, there is less competition among these stages for the small hosts in this scenario. However, in the months of July, August, and September, all the small host capacity is used, indicating a strong competition among larvae (Appendix S1: Figures S42–S44). Note that in this scenario, only larvae use their entire available host capacity during these critical months.

## DISCUSSION

Ixodid ticks play an important role as vectors for various zoonotic pathogens that are emerging due to global change (Jones et al., 2008). Understanding the underlying mechanisms behind seasonal numbers of questing ticks, with the close links to disease hazard (Ostfeld et al., 2006), is therefore an important modeling task (Dobson et al., 2011). Using a new seasonal matrix model, we found that the tick population size is primarily limited by availability of small rather than large hosts, as increasing the large hosts beyond a certain level had no further effect. The level of small host utilization by larvae and nymphs affected competition for small hosts in critical months when host availability was low compared with the demand. An intermediate value of the small host utilization in larvae supports the largest tick population sizes, while the small host utilization by nymphs had a smaller effect. Without seasonal host availability, the total small host capacity was used for a longer duration of time, due to increased competition between larvae and nymphs. In the southern scenario, generation time was much shorter (2.2 years) than in the baseline northern scenario (3.7 years), and an earlier peak availability of small hosts enabled more larvae to feed during their peak questing season. Gathering empirical estimates for all parameters is challenging for any tick dynamics model, and we discuss limitations in terms of uncertainty, in particular regarding the levels of small host utilization by larvae and nymphs, host capacity, and density‐dependent regulation of the tick population.

Many stages of the tick life cycle remain hidden and cannot be directly observed in the field (Dobson, 2014). The visible questing population represents only a small fraction, and traditional methods such as cloth‐lure and dragging techniques often underestimate their actual numbers (Nyrhilä et al., 2020). A key novel aspect of our model is its ability to track the state of individuals with respect to overwintering, feeding, and seasonal timing of feeding and to incorporate effects of these states on individual performance for the next months and year. This feature allows a more detailed understanding of the changing composition of the questing population throughout the season, not only in terms of the main life stages but also with respect to different generations within each life stage, relevant for understanding the transmission of pathogens. While other models have included photoperiod‐induced diapause (e.g., Dobson et al., 2011), to our knowledge, this is the first model for ixodid ticks that includes effects of overwintering and seasonal timing of feeding, affecting the performance of individuals throughout the subsequent year (Grigoryeva & Shatrov, 2022). Delayed life history effects are generally known to impact dynamics across various species and life histories, and it is particularly important to incorporate such effects into models that aim to predict responses to external impacts (Beckerman et al., 2002). In the case of ixodid ticks, individuals that overwinter without feeding will have depleted more resources next summer than ticks that emerge in the same year. This depletion likely affects their survival and potentially their questing behavior as well. Ticks that feed before the summer threshold have a higher probability of molting in the same year, but a lower survival in winter if they have not molted. Ticks that feed after the threshold have a higher probability of survival overwinter, and a low probability of molting until next year. Similarly, ticks that overwinter as fed have a high probability of molting the next summer, and a low probability of survival toward the end of the year. By including such detailed lasting effects, our model obtains a high flexibility to describe a range of scenarios for tick life histories and responses to environmental change.

Arthropod vectors, such as ixodid ticks, are ectotherms that are expanding their distribution toward higher elevations and latitudes under global warming, both in North America and in Europe (Clow et al., 2017; Medlock et al., 2013). Earlier models of *Ixodes* ssp. dynamics have primarily focused on implementing how individual‐level processes of questing and development depend on key environmental drivers such as temperature and saturation deficit (Dobson et al., 2011; Li et al., 2016; Ogden et al., 2005). These models generally assumed a constant host availability and emphasized the direct effects of climate drivers as the primary source of seasonality (Dobson et al., 2011; Ogden et al., 2005). However, it is important to consider that the dynamics of small mammalian hosts in Europe have been altered by both land use and climate change (Cornulier et al., 2013), and there has been a marked increase in the distribution and abundance of large host species such as deer in recent decades (Linnell et al., 2020). Our model allows exploring the additional effects of such changes in the host population on the tick dynamics and seasonal abundance.

Our baseline scenario analysis for *I. ricinus* demonstrated for this model that (1) small hosts are most important in regulating the tick population, unless the density of large hosts is exceptionally low, that (2) the transition of larvae feeding is more important for population regulation than for the feeding of nymphs (and adults), and that (3) the small host utilization of larvae, rather than nymphs, is an important determinant of tick population regulation. Across all scenarios considered, the density regulation primarily occurs through larval feeding in specific months of high activity (Appendix S1: Figures S10–S12, S29–S31, S42–S44). In the baseline northern scenario, a dip in small host feeding by nymphs in June and July (Figure 3b) arises from a combination of factors: (1) a lower number of questing nymphs than of the peak (Figure 3a), (2) a low probability of finding a small host due to their relative scarcity in June (Figure 1), and (3) competition with larvae for available small hosts. Removing the seasonality of small hosts had only small effects on the seasonality of questing ticks in the model (Figure 3a), but it increased the tick population by enabling more ticks to successfully feed during critical questing months (Figure 3b, Appendix S1: Figures S29–S31). In this case, the host capacity is filled through more months than the scenario with a seasonal small host. Thus, the effects of changing host seasonality on tick dynamics are complex, as it not only affects the number of ticks that can feed but also potentially influences density‐dependent regulation. The results for the southern scenario (Appendix S1: Section S3.4) highlight the model's flexibility in capturing diverse tick and host phenology. Unlike the northern scenario, nymphs and larvae in the southern scenario quest at different times with minimal overlap, ensuring that nymphs never compete with larvae for small hosts.

A critical aspect of many population models is the detailed processes by which density dependence is incorporated. In ixodid ticks, there is no clear empirical evidence regarding which stage‐specific vital rates are most affected by density dependence. However, the feeding process on a limited number of hosts is an evident starting point for including such effects. In our model, we assumed a fixed number of available feeding spots per host. Together with seasonally varying host availability, this results in the host capacity being filled during critical months, depending on questing phenology. Additionally, we included scramble competition among nymphs and larvae for small hosts, while large hosts had separate capacities for each of the main tick stages. By contrast, Dobson et al. (2011) incorporated density dependence in a different manner, specifically through survival during the post‐feeding period. In their model, as the number of successfully feeding ticks increased, the survival during the post‐feeding period would decline. This mechanism leads to a more immediate mortality effect of high density during feeding than our model, where ticks that are unable to feed remain in the unfed stage for a longer duration (but indirectly increasing the likelihood of mortality before finding a host). Empirical data on tick load on hosts, as well as other parasites, consistently demonstrate an uneven distribution (Lindsø et al., 2023). Our model does not currently include this complexity, but future developments may expand the model to incorporate variation in tick aggregation on hosts. We also emphasize that both the host availability and feeding capacity were selected without empirical support in our model, as were the probabilities of finding a host. Changing these will alter the size of the modeled tick population, and if the host availability numbers are set too low, the tick population will not be viable.

The relative importance of small and large hosts in regulating tick populations and, consequently, disease hazard has received considerable attention due to contrasting patterns reported (Deblinger et al., 1993; Ostfeld et al., 2006; Rand et al., 2004). Adult ticks rely on large hosts for reproduction, and deer can be important amplifiers of the tick population (James et al., 2013; Ruiz‐Fons & Gilbert, 2010). Our exploration of varying levels of large host availability (Figure 4) suggests that once a certain number of large hosts are available, increasing their availability further has no effect on our modeled tick population. This nonlinear effect of deer density is consistent with other theoretical and empirical findings for *I. scapularis* in North America (Van Buskirk & Ostfeld, 1995) and *I. ricinus* in Europe (Hofmeester et al., 2017; Mysterud et al., 2016). The impact of deer density on tick numbers appears weak or even absent after reaching 5–10 deer per km^2^ or higher (Mysterud et al., 2016). Due to their much lower reproductive rate than that of small mammals, we did not consider seasonality in large hosts in our analyses. Deer typically reproduce only once a year, produce few offspring (red deer: 1; roe deer: 2–3) and have a low maximum annual population growth rate (30%–35% in red deer, >50% in roe deer). However, deer migration may cause considerable seasonality in local densities (van Moorter et al., 2021). Given that deer numbers had a limited role in regulating tick populations in our scenarios (Figure 4), we deemed it beyond the scope of this study to incorporate such effects.

When modeling the transmission of pathogens between vectors and hosts, it is important to consider the life history and seasonality of hosts as well as the vector (Valenzuela‐Sánchez et al., 2021). The current model serves as a good starting point for incorporating such factors, allowing a wide range of life history outcomes to be calculated using matrix model methods (Appendix S1: Section S3.1.2). Currently, our model only includes one small host, which could represent multiple small mammals with similar seasonal variation, typically peaking in the fall (Andreassen et al., 2021). However, the role of birds adds further complexity to the system (Heylen, 2016; Loss et al., 2016). Many bird species are latitudinal migrants, reproducing in the spring and peaking in numbers later in the season. Therefore, while our current model accurately captures the broad seasonal patterns of small hosts, it can easily be extended to include more than one small host to capture differences among them, including finer details in their phenology. While we included seasonality of the small host, the numbers each month were kept constant across years and thus represent only a cartoon of the complex dynamics of small mammals, which can show large annual cycles on top of the seasonal variation (Hansen et al., 1999; Krebs & Myers, 1974). Using a mechanistic agent‐based model Li et al. (2016) included seasonality and multi‐annual cycles in rodent hosts through trigonometric functions. Our current small host model could be expanded in future developments to incorporate such cycles.

Pathogens transmitted by ticks often have a narrower host range than the ticks. Although *I. ricinus* is found on a wide range of mammals and birds (Mysterud et al., 2015), relatively few and common species of vertebrates seem to dominate the transmission dynamics of pathogens in Europe (Hofmeester et al., 2016). When incorporating pathogen dynamics into the model, the inclusion of both competent and incompetent (small) reservoir hosts becomes more important, as their relative abundance can lead to host dilution effects (Keesing & Ostfeld, 2021; Ostfeld & Keesing, 2013). Additionally, the aggregation of ticks on hosts, affecting the potential for co‐feeding transmission (Brunner & Ostfeld, 2008), could then be explicitly modeled. Likewise, different deer species may not vary much in their role as host to ticks, but vary more in their role for transmission of different pathogens (Fabri et al., 2021). Thus, our model serves as a good starting point for incorporating these factors and can be adapted for a broader range of disease systems.

We have presented a new matrix model framework incorporating underlying mechanisms of overwintering, feeding time, and density‐dependent dynamics of feeding, governing the abundance and seasonality of questing ticks important for disease hazard. The model can be used to explore a range of questions regarding the ecology and evolution of ixodid ticks, including comparative analyses of different tick life histories and environments. Our findings from the scenarios considered emphasize the need for a detailed understanding of vector and host ecology in order to capture the underlying mechanisms behind the seasonal numbers of questing ticks. Modeling provides an important tool for assessing the potential impacts of different drivers such as land use and climate change, across species and regions (Rogers & Randolph, 2006).

## AUTHOR CONTRIBUTIONS

Yngvild Vindenes defined the model and did the analyses, with inputs from Atle Mysterud. Both authors contributed to write and revise the manuscript.

## CONFLICT OF INTEREST STATEMENT

The authors declare no conflicts of interest.

## Supporting information


Appendix S1.

## Data Availability

Code (Vindenes & Mysterud, 2024) is available in Figshare at https://doi.org/10.6084/m9.figshare.24961014.
