# Supplementary material for: A seasonal matrix population model for ixodid ticks with complex life histories and limited host availability
Source: Ecology. 2025 Jan 15;106(1):e4511. doi: 10.1002/ecy.4511 (PMC11735348; doi:10.1002/ecy.4511)
Supplement: Supplementary file 1 — Appendix S1. [file ECY-106-e4511-s001.html]

 

 

 

 
 
 


 


 Supporting Information for 

 
 
 
 
 
 
 
 
 
 
 
 
 

 

 
 


 


 

 

 


 


 

 


 


 
 
 
 
 
 

 


 


 Supporting Information for 
 ‘A seasonal matrix population model for ixodid ticks with complex life histories and limited host availability’ 
 Yngvild Vindenes and Atle Mysterud 
 Ecology 

 


   
 The html version of this document presents selected R code, additional analyses and supplementary figures. The R Markdown file including all the R code to reproduce this html document (i.e. all code shown in this html file, plus additional code for analyses and plotting) is provided on figshare:  https://doi.org/10.6084/m9.figshare.24961014 . 
   
 
 Links to tables and figures 
 The following hyperlinks can be used to navigate directly to each supplementary table/figure. 
 
  Table S1 : Overview of tick stages 
  Table S2 : Overview of survival results from observational studies 
  Table S3 : Life history parameters for baseline northern model 
  Table S4 : Life history parameters, constant host seasonality 
  Table S5 : Life history parameters, southern scenario 
 
  Host model and feeding process:  
 
  Figure S1 : Small host vital rates 
  Figure S2 : Host availability 
  Figure S3 : Probability of finding host 
  Figure S4 (fig. 2) : Combined plot, host properties. 
 
  Baseline northern scenario, projections:  
 
  Figure S5 : Baseline parameters 
  Figure S6 : August population over time 
  Figure S7 : Monthly stage size 
 
  Figure S8 : Questing population per month 
  Figure S9 : Numbers feeding per month per host type 
  Figure S10 : Proportion of host capacity used 
  Figure S11 : Proportion of hosts used by each stage 
  Figure S12 : Stage-specific host capacity used 
  Figure S13 : Fed population per month (spring, fall, previous) 
  Figure S14 : Delayed and direct development, including eggs 
  Figure S15 : Delayed and direct development, excluding eggs 
  Figure S16 (fig. 3) : Combined plot, results from baseline 
 
  Baseline northern scenario, life history:  
 
  Figure S17 : Stable stage structure 
  Figure S18 : Reproductive values 
  Figure S19 : Mean remaining lifespan 
  Figure S20 : SD remaining lifespan 
  Figure S21 : Mean lifetime reproductive output per stage 
  Figure S22 : SD lifetime reproductive output per stage 
 
  Vary host parameters:  
 
  Figure S23 (fig. 4) : Varying host levels 
  Figure S24 (fig. 5) : Varying small host utilization 
 
  Constant small host, projections:  
 
  Figure S25 : Projected August population over time 
  Figure S26 : Monthly stage size at equilibrium 
  Figure S27 : Questing population per month 
  Figure S28 : Numbers feeding per month per host type 
  Figure S29 : Proportion available host capacity used 
  Figure S20 : Proportion of hosts used by each stage 
  Figure S31 : Stage-specific host capacity used 
  Figure S32 : Fed population per month (spring, fall, previous) 
  Figure S33 : Delayed and direct development, including eggs 
  Figure S34 : Delayed and direct development, excluding eggs 
  Figure S35 : Combined plot, results with constant hosts. 
 
  Southern scenario:  
 
  Figure S36 : Survival and density independent transitions 
  Figure S37 : Comparison of density independent transitions 
  Figure S38 : Projected August population over time 
  Figure S39 : Monthly stage size 
 
  Figure S40 : Questing population per month 
  Figure S41 : Numbers feeding per month per host type 
  Figure S42 : Proportion available host capacity used 
  Figure S43 : Proportion of hosts used by each stage 
  Figure S44 : Stage-specific host capacity used 
  Figure S45 : Fed population per month (spring, fall, previous) 
  Figure S46 : Delayed and direct development, including eggs 
  Figure S47 : Delayed and direct development, excluding eggs 
  Figure S48 : Combined plot, results compared to northern 
  Figure S49 : Combined plot, results southern scenario 
 
   
 
 
 S1 Setup 
 
 S1.1 Load packages, define stages for plotting 
  #-------------------
#Packages
#-------------------
library(tidyverse)
library(viridis)
library(cowplot) 
library(scales)

#-------------------
#Define stages
#------------------- 
stages <- c("EGG", "EGP", #Egg stages
            "LU",  "LUP", "LFS", "LFF", "LFP", #Larvae stages
            "NU",  "NUP", "NFS", "NFF", "NFP", #Nymph stages
            "AU",  "AUP", "AFS", "AFF", "AFP") #Adult stages

k <- length(stages)  

#-------------------
#Set colors for plotting
#-------------------
colorELNA <- c("#E0C56A", "#B98EDF", "#6FBF66", "#CD7A53") 
color.stages <- c(rep(colorELNA[1],2), rep(colorELNA[2],5), rep(colorELNA[3],5), rep(colorELNA[4],5))  
   
 
 
 S1.2 R functions for life history calculations 
 The functions for calculating the offspring mixing distribution, mean and variance in lifespan, and mean and variance of lifetime reproduction are from Hernandez  et al.  (2024), adapted to the notation used here. 
  #Lambda, stable structure and reproductive values based on eigen analysis:
uvlambda <- function(MatA){
  ev <- eigen(MatA)
  tev <- eigen(t(MatA))
  lmax <- which.max(Re(ev$values))
  U <- ev$vectors
  V <- tev$vectors
  u <- as.matrix(abs(Re(U[, lmax]))/sum(abs(Re(U[, lmax]))))
  u <- u/(sum(u))
  v <- as.matrix(abs(Re(V[, lmax])))
  v <- v/sum(u*v )
  v <- ifelse(u*v <= 0, 0, v)
  v2 <- v/v[1]
  return(list("lambda"=max(Re(ev$values)),"u"=as.vector(u),"v"=as.vector(v),"v2"=as.vector(v2)))
}
 
#Net reproductive rate (mean lifetime reproductive output):
R0function  <-  function(MatF, MatU){
     k <- dim(MatF)[1] 
     N <- solve(diag(1,k,k)-MatU)
     MatR <- MatF%*%N
     resR<- uvlambda(MatR)
     resR$lambda
}

#Generation time (mean age of mothers at stable distribution, weighted by offspring):
GenTime  <-  function(MatA, MatF){
  res <- uvlambda(MatA=MatA)
  lam <- res$lambda
  u <- res$u
  v <- res$v
  lam/(t(v)%*%MatF%*%u)    
}

#----------------------
# The following functions are adapted from  Hernandez, C. M., Ellner, S. P., Snyder, R. E., & Hooker, G. (2024). Supplemental code and data for Hernandez et al. "The natural history of luck: A synthesis study of structured population models". https://doi.org/10.5281/zenodo.10527715.


#Mixing distribution of offspring (proportion in each stage). 
mixing_distro<- function(MatA, MatF){
  stable_dist <- uvlambda(MatA = MatA)$u
  offspring<- MatF%*%stable_dist
  offspring<- offspring/sum(offspring)
  return(offspring)
}

#Mean lifespan
mean_lifespan<- function(MatU, mixdist=NULL){
  k <- dim(MatU)[1]
  expLCond_z <- rep(1, k)%*%solve(diag(1,k,k)-MatU)
  if(!is.null(mixdist)){
    expL <- expLCond_z%*%mixdist
    return(expL)
  } else{
    return(expLCond_z)
  }
}

# Calculate the variance in lifespan:
# note: this calculates the variance in the number of time steps!
var_lifespan<- function(MatU, mixdist=NULL){
  k <- dim(MatU)[1]
  N <- solve(diag(1,k,k)-MatU)
  expLCond_z <- mean_lifespan(MatU, mixdist = NULL)
  eT <- matrix(data=1, ncol=k, nrow=1) # column vector of 1's
  varLCond_z<- eT %*% (2*N%*%N - N) - (expLCond_z)^2
  if(is.null(mixdist)){
    return(varLCond_z)
  } else{
    # variance in lifespan due to differences along trajectories:
    varL_within<- varLCond_z %*% mixdist 
    # variance in lifespan due to differences among starting states:
    varL_between<- t(mixdist)%*%t(expLCond_z^2) - (t(mixdist)%*%t(expLCond_z))^2
    # total variance in lifespan, given the mixing distribution:
    varL<- varL_within + varL_between
    return(varL)
  }
}

# Calculate the expected value of (remaining) lifetime reproductive success
mean_LRO<- function(MatU, MatF, mixdist=NULL){
  k <- dim(MatU)[1]
  expRCond_z<- rep(1,k)%*%MatF%*%solve(diag(1,k,k)-MatU)
  if(!is.null(mixdist)){
    expR <- expRCond_z%*%mixdist
    return(expR)
  } else{
    return(expRCond_z)
  }
}

# Calculate the variance of (remaining) lifetime reproductive success
var_LRO<- function(MatU, MatF, repro_var = 'poisson', mixdist=NULL ){
  k <- dim(MatU)[1]
  # Take the sum of offspring:
  betabar<- colSums(MatF) #betabar is the average offspring production
  # Variance in per-capita number of offspring per time step:
  if (repro_var %in% c("poisson", "Poisson")){
    sigsqb<- betabar # poisson
  } else if (repro_var %in% c("Bernoulli", "bernoulli")){
    sigsqb<- betabar*(1 - betabar) # bernoulli
  } else if (repro_var %in% c("fixed", "Fixed")){
    sigsqb<- 0
  }
  expRCond_z <- mean_LRO(MatU, MatF)
  rbarPib = expRCond_z %*% MatU
  r2 = (sigsqb + (betabar)^2 + 2*betabar*rbarPib) %*% solve(diag(1,k,k)-MatU)
  varRCond_z = (r2 - expRCond_z^2)
  if(is.null(mixdist)){
    return(varRCond_z)
  } else{
    # variance in LRO due to differences along trajectories:
    varR_within <- varRCond_z%*%mixdist 
    # variance in LRO due to differences among starting states:
    varR_between <- t(mixdist)%*%t(expRCond_z^2) - (t(mixdist)%*%t(expRCond_z))^2
    # total variance in LRO, given the mixing distribution:
    varR <- varR_within + varR_between
    return(varR)
  }
}  
  
  
 
 
 
 S2 Model definition 
 
 S2.1 Overview of tick stages 
 
  Table S1:  Overview of tick stages in the model. Density and host dependence occurs through the feeding process (section 2.1) defining transition probabilities from unfed to fed stages. 
 
 
 Number 
 Stage 
 Description 
 
 
 
 
 1 
 EGG 
 Egg laid current year 
 
 
 2 
 EGP 
 Egg laid previous year 
 
 
 3 
 LU 
 Unfed larva hatched current year 
 
 
 4 
 LUP 
 Unfed larva hatched previous year 
 
 
 5 
 LFS 
 Larva fed in spring current year 
 
 
 6 
 LFF 
 Larva fed in fall current year 
 
 
 7 
 LFP 
 Larva fed previous year 
 
 
 8 
 NU 
 Unfed nymph emerged current year 
 
 
 9 
 NUP 
 Unfed nymph emerged previous year 
 
 
 10 
 NFS 
 Nymph fed in spring current year 
 
 
 11 
 NFF 
 Nymph fed in fall current year 
 
 
 12 
 NFP 
 Nymph fed previous year 
 
 
 13 
 AU 
 Unfed adult emerged current year 
 
 
 14 
 AUP 
 Unfed adult emerged previous year 
 
 
 15 
 AFS 
 Adult fed in spring current year 
 
 
 16 
 AFF 
 Adult fed in fall current year 
 
 
 17 
 AFP 
 Adult fed previous year 
 
 
 
   
 
 
 S2.1 Host availability and feeding 
 We assume that the feeding of ticks is limited by the availability of small hosts ( \(S_i\) ) and large hosts ( \(L_i\) ). Adult ticks in the model must feed on a large host, while nymphs and larvae can feed on both large and small hosts. Larvae are assumed to utilize small hosts with a probability of 0.9 in the baseline model, while nymphs are more generalists with a small host utilization of 0.5. We assume that large hosts have small or no seasonal fluctuations and thus are available throughout the year. 
   
 
 S2.1.1 Small host model 
 To describe the seasonal availability of small hosts, we add a simple model for the monthly dynamics of a generic small mammal (e.g., similar to bank vole,  Clethrionomys glareolus ), where survival and fecundity have a constant value each month leading to a seasonal cycle, but the population is reset every year in January to the same starting population. The model assumes a post-reproductive census where individuals are counted in the beginning of each month, right after reproduction. It separates between offspring and adults, where only adults are available to ticks and counted in the host availability numberts. Offspring are assumed to be unavailable for ticks due to being in the burrow (lactation time approximately 16 days; Innes and millar, 1994), and we assume that individuals can reproduce for the first time at the age of two months. 
 Survival of bank voles is typically higher in winter than in summer (Crespin  et al. , 2002). We assume for the baseline model that monthly survival takes values from 0.9 in winter to 0.6 in summer (Fig. S1), within the range reported by Crespin  et al.  (2002) for a population in the Netherlands, when translating seven-day survival to monthly survival. 
 Fecundity values for the baseline model were set based on information from Nyholm and Meurling (1979) for a northern and southern Swedish population, where we use the southern one for our model. The average litter size in the model is set to 5, close to the reported value of 5.2 by Nyholm and Meurling (1979). Litter size can range from 1-8 (Nyholm and Meurling, 1979). The proportion of females reproducing is set to 0.1 in May, as Nyholm and Meurling (1979) observed some pregnant females in this month and the gestation time is around 21 days (Innes and Millar, 1994). We assume the proportion is higher in the months of June (0.8), July (0.9) and August (0.8), and reduced to 0.6 for September, with no reproduction for the rest of the year (Nyholm and Meurling, 1979). 
 In the constant scenario without seasonality, we remove the seasonality in both survival and fecundity. In this scenario we assume a constant proportion of 0.8 of females reproducing per month, and a constant survival of 0.5 (exact values do not matter here, as long as the resulting host availability is constant across months).Note that this is a purely hypothetical scenario used for comparison in the analyses, as some degree of seasonality is likely present in all bank vole populations. 
 In the alternative scenario for southern Europe, we assume reproduction starts much earlier in the year, in February, and ends in summer (last reproductive month June) when plant growth stops due to drought (Andreassen  et al. , 2021). Survival is assumed to be more constant throughout the season, and reproduction somewhat lower since the reproductive season is longer. 
   
   
 
 
Figure S1: Small host vital rates, used to generate seasonal numbers of available small hosts (fig. S2).
 
 
   
   
 
 
Figure S2: Available small hosts and large hosts per month in the model, for three different scenarios. The small host availability is seasonal in the baseline model and the southern scenario. The large host availability is constant in all scenarios (500).
 
 
   
 
 
 S2.1.2 Probability of finding a host 
 The probability that a questing tick finds a host depends on the tick instar, the host type, and the current number of available hosts. We define it as a logistic function, as described in the main text. The maximum probability depends on tick stage and host type. Note that here we set the probability that an adult finds a small host to zero, but in the model the small host utilization by adults is 0 so the value of the probability here does not matter. If this is modified, the probability of finding a small host by adults should also be adjusted. 
 The choice of a logistic model builds on the notions that i) the probability of finding a host should be zero when there are no hosts available, ii) the probability of finding a host should increase with the number of available hosts, and iii) the probability of finding a host should level out at a certain level of host availability. Furthermore, we assume that due to higher mobility adults are better at finding a host than nymphs and larvae, and nymphs are better than larvae. To our knowledge there is no available empirical data to estimate the parameters of the host finding probability functions, but it is not meant to predict precise quantitative numbers but rather qualitative patterns in the feeding process. 
  p.find.host.small <- function(hosts, max.prob = 0.8, ks =7e-4, H0 = 1500){
  max.prob * (1+exp(-ks*(hosts-H0)))^(-1)
}

p.find.host.large <- function(hosts, max.prob = 0.8, kL =.005, H0 = 100){
 max.prob * (1+exp(-kL*(hosts-H0)))^(-1)
}  
   
   
 
 
Figure S3: The probability that a questing tick finds a small or large host as a function of host availability, tick instar (larva, nymph or adult) and host type (small or large). Vertical lines indicate the baseline starting value of small hosts in January (left), and the baseline constant values of large hosts (right).
 
 
   
 
 
 S2.1.3 Combined plot 
   
 
 
Figure S4: Figure 2 in main text.
 
 
   
 
 
 
 S2.2 Tick model 
 
 S2.2.1 Baseline parameters 
 The baseline model represents a ‘northern’ tick life history with high survival rates, slow development rates and short questing season. This is compared to a ‘southern’ life history below with lower survival, faster development and longer questing season. 
   
 
 Survival 
 In general, the probability of survival over  \(t\)  days is given by 
  \[S(t)=\exp(-mt),\]  
 where  \(m\)  is the daily mortality rate. Table S2 below shows monthly survival rates calculated from the daily mortality rates reported in Table 1 of Randolph (2004) and from the results of Grigoryeva and Shatrov (2022), for different tick stages. These estimates derive from observation studies in containers from the Czech Republique (Daniel  et al.  1976), from Ireland (Gray 1981), and from Russia (Grigoryeva and Shatrov 2022). As Randolph (2004) discusses, the mortality rates reported from observational studies using containers are generally much lower than the theoretical requirement to maintain a population over time. Among other factors, such studies do not measure mortality from predation and during feeding. 
   
 
  Table S2:  Daily survival and monthly survival estimates based on table 1 from Randolph (2004) and results from Grigoryeva and Shatrov (2022), using our notation for stages and adding monthly survival probabilities assuming 30 days per month. Period refers to the observation period in the study used to calculate the mortality rate. 
 
 
 
 
 
 
 
 
 
 
 Stage 
 Period 
 DailyMortalityRate 
 MonthlySurvival 
 Location 
 Reference 
 
 
 
 
 LU/LUP 
 July-Oct 
 0.00670 
 0.8179 
 Czech Republic 
 Daniel  et al.  (1976) 
 
 
 LU/LUP 
 Oct-Apr 
 0.00180 
 0.9474 
 Czech Republic 
 Daniel  et al.  (1976) 
 
 
 LU/LUP 
 Oct-Apr 
 0.00077 
 0.9772 
 North east Russia 
 Grigoryeva and Shatrov (2022) 
 
 
 LFF/LFP 
 Nov-Apr 
 0.00200 
 0.9418 
 Czech Republic 
 Daniel  et al.  (1976) 
 
 
 LFF/LFP 
 Oct-Aug 
 0.00420 
 0.8816 
 Czech Republic 
 Daniel  et al.  (1976) 
 
 
 LFF/LFP 
 Aug-Aug 
 0.00180 
 0.9474 
 Ireland 
 Gray (1981) 
 
 
 LFF/LFP 
 Oct-Apr 
 0.00060 
 0.9822 
 North east Russia 
 Grigoryeva and Shatrov (2022) 
 
 
 LFS 
 May-Aug 
 0.00130 
 0.9618 
 Czech Republic 
 Daniel  et al.  (1976) 
 
 
 LFS 
 May-Sept 
 0.00110 
 0.9675 
 Ireland 
 Gray (1981) 
 
 
 NU/NUP 
 Oct-Apr 
 0.00110 
 0.9675 
 North east Russia 
 Grigoryeva and Shatrov (2022) 
 
 
 NU/NUP 
 Nov-Apr 
 0.00210 
 0.9389 
 Czech Republic 
 Daniel  et al.  (1976) 
 
 
 NFF/NFP 
 Nov-Apr 
 0.00020 
 0.9940 
 Czech Republic 
 Daniel  et al.  (1976) 
 
 
 NFF/NFP 
 Aug-Aug 
 0.00080 
 0.9763 
 Czech Republic 
 Daniel  et al.  (1976) 
 
 
 NFF/NFP 
 Oct-Sep 
 0.00060 
 0.9822 
 Ireland 
 Gray (1981) 
 
 
 NFF/NFP 
 Oct-Apr 
 0.00080 
 0.9763 
 North east Russia 
 Grigoryeva and Shatrov (2022) 
 
 
 NFS 
 Apr-Aug 
 0.00060 
 0.9822 
 Czech Republic 
 Daniel  et al.  (1976) 
 
 
 NFS 
 May-Aug 
 0.00130 
 0.9618 
 Ireland 
 Gray (1981) 
 
 
 AU/AUP 
 Nov-Apr 
 0.00140 
 0.9589 
 Czech Republic 
 Daniel  et al.  (1976) 
 
 
 AU/AUP 
 Oct-Apr 
 0.00030 
 0.9910 
 North east Russia 
 Grigoryeva and Shatrov (2022) 
 
 
 AFF/AFP 
 Nov-Jul 
 0.00120 
 0.9646 
 Czech Republic 
 Daniel  et al.  (1976) 
 
 
 AFF/AFP 
 Sep-Aug 
 0.00500 
 0.8607 
 Ireland 
 Gray (1981) 
 
 
 AFF/AFP 
 Oct-Apr 
 0.00010 
 0.9970 
 North east Russia 
 Grigoryeva and Shatrov (2022) 
 
 
 AFS 
 May-Sep 
 0.00009 
 0.9973 
 Ireland 
 Gray (1981) 
 
 
 
   
 The data frame ‘survival’ contains our chosen monthly survival probabilities for each tick stage and month in our baseline model representing a northern European tick population. We assume that overwintered individuals have the same survival as individuals hatched in the same year within each main stage (egg, larvae, nymph, adult) in the beginning of each season, but that survival of overwintered individuals progressively declines from summer to reach 0 in fall. We have chosen somewhat lower values of survival than those reported from the observation studies in table S1. Our values are still high enough to maintain high tick population growth rates when the tick population is small and host availability is high. Eventually the tick population will be regulated by the host availability to have an average annual population growth rate of 1. 
   
  survival <- data.frame(
  "Month"= month.abb, 
  "MonthNum"= 1:12, 
  #----------------
  "EGG" = rep(0.91, 12), 
  "EGP" = c(rep(0.91, 5), .8, .7, .6, .3, rep(0, 3)),
  #----------------
  "LU"  = rep(0.92, 12),  
  "LUP" = c(rep(.92, 5), .8, .7, .6, .5, .4, 0, 0), 
  "LFS" = c(rep(.95, 8), .5, .2, 0, 0),
  "LFF" = rep(.95, 12), 
  "LFP" = c(rep(.95, 6), .7, .6, .5, 0, 0, 0), 
  #----------------
  "NU"  = rep(.95, 12),
  "NUP" = c(rep(.95, 7), .8, .6, .5, 0, 0),
  "NFS" = c(rep(.97, 8),.3, .2, 0, 0),
  "NFF" = rep(.97, 12),  
  "NFP" = c(rep(.97, 8), .7, .1, 0, 0), 
  #---------------
  "AU"  = c(rep(0.95, 12)),
  "AUP" = c(rep(.95, 9),   .8,   .4, 0),
  "AFS" = c(rep(.99, 9), .3, 0, 0),
  "AFF" = c(rep(.99, 12)),
  "AFP" = c(rep(.99, 8), .3, .1,0, 0)
  )  
   
 While the differences in survival between stages may seem small on a monthly scale, the accumulated difference over several months is large: A monthly survival probability of 0.91 means that the probability of survival over 10 months will be  \(0.91^{10}\approx 0.39\) . Similarly, a monthly survival probability of 0.95 yields a 10-month survival probability of  \(0.95^{10}\approx 0.60\) , while a monthly survival of 0.99 yields a 10-month survival probability of  \(0.99^{10}\approx 0.90\) . 
 Considering that a female on average produces around 1500 eggs, a life history that maintains the population stable over time requires that only 1 in 750 (female) individuals survive to reproduce on average. Thus, it is expected that accumulated survival over the entire life cycle is very low. For three years (36 months) this would correspond to an average monthly survival probability of 
  \[\exp(\ln(1/750)/36)≈0.832.\] 
This value is only a rough estimate and represents the combined probability of survival and transition through all stages.The survival probability is generally higher during diapause (protected by the leaf litter) and lower during stages of higher exposure (questing and feeding). 
   
 
 
 Probabilities of hatching, molting and questing 
 The duration and peak of each of these processes in the baseline population were informed by information n Grigoryeva and Shatrov (2022), Randolph (2004), Gray  et al.  (2016) Kahl and Gray (2023), as explained below. In addition we tuned the magnitude of the parameters during initial model development to obtain a tick model that met these criteria: 1) A viable population (approaching a stable seasonal cycle), 2) a generation time between 3 and 4 years, and 3) a population of questing larvae being approximately an order of magnitude larger than the population of questing nymphs. 
 In the study of Grigoryeva and Shatrov (2022), around 86% of the eggs hatched to larvae, with hatching between June and October. Larvae can hatch from eggs laid earlier in the year by females who fed the year before, or females that fed earlier in the same year. Eggs laid late in the season may also overwinter and hatch next year. In our model we assume that most eggs hatch in July and August, whether they are overwintered or not. Overwintered eggs are assumed to have progressive reduction in survival probability for each month after June, reaching 0 by October so that no eggs can survive two winters in the model. 
 The tick development to the next stage after a blood meal (including processes of digestion, molting, and hardening after the molt) depends on temperature and light conditions (Randolph 2004; Gray  et al.  2016; Kahl and Gray 2023). Conditions of decreasing daylength induces diapause, which delays onset of the development process until next summer (Gray  et al.  2016). The development only occurs in the warm season, between June and October in central and northern Europe (Kahl and Gray 2023). Development rates increase with temperature (Randolph 2004). In the study of Grigoryeva and Shatrov (2022), of the fed larvae around 77% survived and completed the development to nymphs, while around 85% of fed nymphs survived and developed to adults. Larvae feeding in early summer may molt to nymphs in the late summer of the same year, while larvae feeding in late summer and fall will overwinter as fed and molt to nymphs in late summer of the next year (Grigoryeva and Shatrov 2022). In our model we assume a peak molting probability of larvae and numphs in August. The combined probability of 
 The probability of questing for unfed individuals depends on stage and on environmental factors like saturation deficit and temperature (Randolph 2004). This leads to a seasonal pattern with higher activity in summer months for northern and central Europe. 
 The data frame ‘develop’ contains the density independent probabilities of hatching (for eggs), questing (for unfed stages), molting (fed stages of larvae and nymphs), and reproducing and dying (adult fed females). These probabilities define all transitions except the transitions from unfed to fed stages, which depend on the feeding process described in section S2.1. 
  develop <- data.frame(
  "Month"= month.abb, 
  "MonthNum"= 1:12, 
  "EGG" = c(rep(0, 4), .01, .1, .4, .4, .1, .01, 0, 0), #hatching (LU next month) 
  "EGP" = c(rep(0, 4), .01, .1, .4, .4, .1, .01, 0, 0), #hatching (LU next month)
  #----------------
  "LU"  = c(rep(0, 6),  .01, .05, .02, .01,0,0), #questing (most will wait until next year)
  "LUP" = c(rep(0, 4), .05, .3, .4, .2, .1, .01, 0, 0), #questing 
  "LFS" = c(rep(0,6), .1, .3, .2, .01, 0, 0) , #molting (NU next month)
  "LFF" = c(rep(0,8), 0, 0, 0, 0),   #molting (NU next month)
  "LFP" = c(rep(0,5),.05, .2, .3, .1, 0, 0, 0) , #molting (NU next month)
  #----------------
  "NU"  = c(rep(0, 6), .05, .1, .07,.05,.01,0), #questing (most will wait until next year)
  "NUP" = c(rep(0,3), .3, .6, .5, .3, .2, .01, .01,0,0), #questing 
  "NFS" = c(rep(0,6), .1, .3, .2, .01,  0, 0), #molting (AU next month)
  "NFF" = rep(0, 12), #molting (AU next month)
  "NFP" = c(rep(0,5),.05, .1, .5, .4, .05,  0, 0), #molting (AU next month)
  #---------------
  "AU"  = c(rep(0, 6),  .05, .1, .07, .05, .01,0), #questing (most will wait until next year)
  "AUP" = c(rep(0, 3), .2, .5, .6, .5, .2, 0.03, .01, 0, 0), #questing
  "AFS" = c(rep(0, 4),  0, .3, .4, .3, .1, 0, 0,0), #reproduce next month  (and die)
  "AFF" = c(rep(0, 7), .01, .05, 0,0,0), #reproduce next month  (and die)
  "AFP" = c(rep(0, 3), .1,  .2, .7, .7, .5, .3, 0, 0,0) #reproduce next month (and die)
)  
   
   
 
 
Figure S5: Baseline values of survival and density independent probabilities of hatching (eggs), questing (unfed stages), molting (fed stages of larvae and nymphs) and reproducing / dying (adult fed stages). The vertical grey lines indicate the threshold month for spring and fall feeding (July).
 
 
   
 
 
 Fecundity 
 The probability that a fed adult female will reproduce will also affect survival, since reproduction is fatal. We assume each reproducing female lays 1500 eggs, reflecting a typical average value (Gray 1981, Grigoryeva and Shatrov 2002). The egg laying process of  I. ricinus  depends on temperature, and takes around 30 days at 15°C (Dautel and Knülle, 2010). 
   
 
 
 
 S2.2.2 Projection matrices 
 The projection matrix determining transitions of ticks between stages from one month to the next is depending on month and on the current density of ticks and hosts. In the baseline scenario the threshold month for spring/fall feeding is set to July. 
 From January to June ( \(j=1,...,6\) ), the projection matrix for month  \(j\)  at time  \(t\)  is given by 
   
    \[
\mathbf{A}_{tj}(\mathbf{n_t},S_j,L_j) = \begin{array}{c|ccccccccccccccccc}
&\text{EGG}&\text{EGP}&\text{LU}&\text{LUP}&\text{LFS}&\text{LFF}&\text{LFP}&\text{NU}&\text{NUP}&\text{NFS}&\text{NFF}&\text{NFP}&\text{AU}&\text{AUP}&\text{AFS}&\text{AFF}&\text{AFP} \\
\hline\\
\text{EGG}  & s_{1j}(1-t_{1j})&0&0&0&0&0&0&0&0&0&0&0&0&0&0.5s_{15j}t_{15j}b_{15j}&0.5s_{16j}t_{16j}b_{16j}&0.5s_{17j}t_{17}b_{17j} \\
\text{EGP}  & 0&s_{2j}(1-t_{2j})&0&0&0&0&0&0&0&0&0&0&0&0&0&0&0 \\
\text{LU}   & s_{1j}t_{1j}&s_{2j}t_{2j}&s_{j3}(1-t_{3j}(\mathbf{n_t},S_t,L_t))&0&0&0&0&0&0&0&0&0&0&0&0&0&0 \\
\text{LUP}  &0&0&0&s_{4j}(1-t_{4j}(\mathbf{n_t},S_t,L_t))&0&0&0&0&0&0&0&0&0&0&0&0&0 \\
\text{LFS}  &0&0&s_{3j}t_{3j}(\mathbf{n_t},S_t,L_t)&s_{4j}t_{4j}(\mathbf{n_t},S_t,L_t)&s_{5j}(1-t_{5j})&0&0&0&0&0&0&0&0&0&0&0&0 \\
\text{LFF}  &0&0&0&0&0&s_{6j}(1-t_{6j})&0&0&0&0&0&0&0&0&0&0&0 \\
\text{LFP}  &0&0&0&0&0&0&s_{7j}(1-t_{7j})&0&0&0&0&0&0&0&0&0&0 \\
\text{NU}   &0&0&0&0&s_{5j}t_{5j}&s_{6j}t_{6j}&s_{7j}t_{7j}&s_{8j}(1-t_{8j}(\mathbf{n_t},S_t,L_t))&0&0&0&0&0&0&0&0&0 \\
\text{NUP}  &0&0&0&0&0&0&0&0&s_{9j}(1-t_{9j}(\mathbf{n_t},S_t,L_t))&0&0&0&0&0&0&0&0 \\
\text{NFS}  &0&0&0&0&0&0&0&s_{8j}t_{8j}(\mathbf{n_t},S_t,L_t)&s_{9j}t_{9j}(\mathbf{n_t},S_t,L_t)&s_{10j}(1-t_{10j})&0&0&0&0&0&0&0 \\
\text{NFF}  &0&0&0&0&0&0&0&0&0&0&s_{11j}(1-t_{11j})&0&0&0&0&0&0 \\
\text{NFP}  &0&0&0&0&0&0&0&0&0&0&0&s_{12j}(1-t_{12j})&0&0&0&0&0 \\
\text{AU}   &0&0&0&0&0&0&0&0&0&s_{10j}t_{10j}&s_{11j}t_{11j}&s_{12j}t_{12j}&s_{13j}(1-t_{13j}(\mathbf{n_t},S_t,L_t))&0&0&0&0 \\
\text{AUP}  &0&0&0&0&0&0&0&0&0&0&0&0&0&s_{14j}(1-t_{14j}(\mathbf{n_t},S_t,L_t))&0&0&0 \\
\text{AFS}  &0&0&0&0&0&0&0&0&0&0&0&0&s_{13j}t_{13j}(\mathbf{n_t},S_t,L_t)&s_{14j}t_{14j}(\mathbf{n_t},S_t,L_t)&s_{15j}(1-t_{15j})&0&0 \\
\text{AFF}  &0&0&0&0&0&0&0&0&0&0&0&0&0&0&0&s_{16j}(1-t_{16j})&0 \\
\text{AFP}  &0&0&0&0&0&0&0&0&0&0&0&0&0&0&0&0&s_{17j}(1-t_{17j})\end{array}.
\]  
   
   
 Note that the non-zero elements only indicate possible transitions. For instance, there will not be any fall fed individuals in the population in these these months, so it does not matter what the transition proabilities are for these stages in these months. 
 From July to November ( \(j=7,...,11\) ), the projection matrix for month  \(j\)  and time  \(t\)  is given by 
   
    \[
\mathbf{A}_{tj}(\mathbf{n_t},S_j,L_j) = \left[\begin{array}{c|ccccccccccccccccc}
&\text{EGG}&\text{EGP}&\text{LU}&\text{LUP}&\text{LFS}&\text{LFF}&\text{LFP}&\text{NU}&\text{NUP}&\text{NFS}&\text{NFF}&\text{NFP}&\text{AU}&\text{AUP}&\text{AFS}&\text{AFF}&\text{AFP} \\
\hline\\
\text{EGG}  & s_{1j}(1-t_{1j})&0&0&0&0&0&0&0&0&0&0&0&0&0&0.5s_{15j}t_{15j}b_{15j}&0.5s_{16j}t_{16j}b_{16j}&0.5s_{17j}t_{17}b_{17j} \\
\text{EGP}  & 0&s_{2j}(1-t_{2j})&0&0&0&0&0&0&0&0&0&0&0&0&0&0&0 \\
\text{LU}   & s_{1j}t_{1j}&s_{2j}t_{2j}&s_{j3}(1-t_{3j}(\mathbf{n_t},S_t,L_t))&0&0&0&0&0&0&0&0&0&0&0&0&0&0 \\
\text{LUP}  &0&0&0&s_{4j}(1-t_{4j}(\mathbf{n_t},S_t,L_t))&0&0&0&0&0&0&0&0&0&0&0&0&0 \\
\text{LFS}  &0&0&0&0&s_{5j}(1-t_{5j})&0&0&0&0&0&0&0&0&0&0&0&0 \\
\text{LFF}  &0&0&s_{3j}t_{3j}(\mathbf{n_t},S_t,L_t)&s_{4j}t_{4j}(\mathbf{n_t},S_t,L_t)&0&s_{6j}(1-t_{6j})&0&0&0&0&0&0&0&0&0&0&0 \\
\text{LFP}  &0&0&0&0&0&0&s_{7j}(1-t_{7j})&0&0&0&0&0&0&0&0&0&0 \\
\text{NU}   &0&0&0&0&s_{5j}t_{5j}&s_{6j}t_{6j}&s_{7j}t_{7j}&s_{8j}(1-t_{8j}(\mathbf{n_t},S_t,L_t))&0&0&0&0&0&0&0&0&0 \\
\text{NUP}  &0&0&0&0&0&0&0&0&s_{9j}(1-t_{9j}(\mathbf{n_t},S_t,L_t))&0&0&0&0&0&0&0&0 \\
\text{NFS}  &0&0&0&0&0&0&0&0&0&s_{10j}(1-t_{10j})&0&0&0&0&0&0&0 \\
\text{NFF}  &0&0&0&0&0&0&0&s_{8j}t_{8j}(\mathbf{n_t},S_t,L_t)&s_{9j}t_{9j}(\mathbf{n_t},S_t,L_t)&0&s_{11j}(1-t_{11j})&0&0&0&0&0&0 \\
\text{NFP}  &0&0&0&0&0&0&0&0&0&0&0&s_{12j}(1-t_{12j})&0&0&0&0&0 \\
\text{AU}   &0&0&0&0&0&0&0&0&0&s_{10j}t_{10j}&s_{11j}t_{11j}&s_{12j}t_{12j}&s_{13j}(1-t_{13j}(\mathbf{n_t},S_t,L_t))&0&0&0&0 \\
\text{AUP}  &0&0&0&0&0&0&0&0&0&0&0&0&0&s_{14j}(1-t_{14j}(\mathbf{n_t},S_t,L_t))&0&0&0 \\
\text{AFS}  &0&0&0&0&0&0&0&0&0&0&0&0&0&0&s_{15j}(1-t_{15j})&0&0 \\
\text{AFF}  &0&0&0&0&0&0&0&0&0&0&0&0&s_{13j}t_{13j}(\mathbf{n_t},S_t,L_t)&s_{14j}t_{14j}(\mathbf{n_t},S_t,L_t)&0&s_{16j}(1-t_{16j})&0 \\
\text{AFP}  &0&0&0&0&0&0&0&0&0&0&0&0&0&0&0&0&s_{17j}(1-t_{17j})\end{array} \right].
\]  
   
   
 For December ( \(j=12\) ), the projection matrix at time  \(t\)  is given by 
   
   
  \[
\mathbf{A}_{tj}(\mathbf{n_t},S_j,L_j) = \left[\begin{array}{c|ccccccccccccccccc}
&\text{EGG}&\text{EGP}&\text{LU}&\text{LUP}&\text{LFS}&\text{LFF}&\text{LFP}&\text{NU}&\text{NUP}&\text{NFS}&\text{NFF}&\text{NFP}&\text{AU}&\text{AUP}&\text{AFS}&\text{AFF}&\text{AFP} \\
\hline\\
\text{EGG}  & 0&0&0&0&0&0&0&0&0&0&0&0&0&0&0&0&0\\
\text{EGP}  & s_{1j}(1-t_{1j})&s_{2j}(1-t_{2j})&0&0&0&0&0&0&0&0&0&0&0&0&0.5s_{15j}t_{15j}b_{15j}&0.5s_{16j}t_{16j}b_{16j}&0.5s_{17j}t_{17}b_{17j} \\
\text{LU}   &0&0&0&0&0&0&0&0&0&0&0&0&0&0&0&0&0 \\
\text{LUP}  & s_{1j}t_{1j}&s_{2j}t_{2j}&s_{j3}(1-t_{3j}(\mathbf{n_t},S_t,L_t))&s_{4j}(1-t_{4j}(\mathbf{n_t},S_t,L_t))&0&0&0&0&0&0&0&0&0&0&0&0&0 \\
\text{LFS}  &0&0&0&0&0&0&0&0&0&0&0&0&0&0&0&0&0 \\
\text{LFF}  &0&0&0&0&0&0&0&0&0&0&0&0&0&0&0&0&0 \\
\text{LFP}  &0&0&s_{3j}t_{3j}(\mathbf{n_t},S_t,L_t)&s_{4j}t_{4j}(\mathbf{n_t},S_t,L_t)&s_{5j}(1-t_{5j})&s_{6j}(1-t_{6j})&s_{7j}(1-t_{7j})&0&0&0&0&0&0&0&0&0&0 \\
\text{NU}  &0&0&0&0&0&0&0&0&0&0&0&0&0&0&0&0&0 \\
\text{NUP}   &0&0&0&0&s_{5j}t_{5j}&s_{6j}t_{6j}&s_{7j}t_{7j}&s_{8j}(1-t_{8j}(\mathbf{n_t},S_t,L_t))&s_{9j}(1-t_{9j}(\mathbf{n_t},S_t,L_t))&0&0&0&0&0&0&0&0 \\
\text{NFS}  &0&0&0&0&0&0&0&0&0&0&0&0&0&0&0&0&0 \\
\text{NFF}  &0&0&0&0&0&0&0&0&0&0&0&0&0&0&0&0&0 \\
\text{NFP}  &0&0&0&0&0&0&0&s_{8j}t_{8j}(\mathbf{n_t},S_t,L_t)&s_{9j}t_{9j}(\mathbf{n_t},S_t,L_t)&s_{10j}(1-t_{10j})&s_{11j}(1-t_{11j})&s_{12j}(1-t_{12j})&0&0&0&0&0 \\
\text{AU}   &0&0&0&0&0&0&0&0&0&0&0&0&0&0&0&0&0 \\
\text{AUP}  &0&0&0&0&0&0&0&0&0&s_{10j}t_{10j}&s_{11j}t_{11j}&s_{12j}t_{12j}&s_{13j}(1-t_{13j}(\mathbf{n_t},S_t,L_t))&s_{14j}(1-t_{14j}(\mathbf{n_t},S_t,L_t))&0&0&0 \\
\text{AFS}  &0&0&0&0&0&0&0&0&0&0&0&0&0&0&0&0&0 \\
\text{AFF}  &0&0&0&0&0&0&0&0&0&0&0&0&9&0&0&0&0 \\
\text{AFP}  &0&0&0&0&0&0&0&0&0&0&0&0&s_{13j}t_{13j}(\mathbf{n_t},S_t,L_t)&s_{14j}t_{14j}(\mathbf{n_t},S_t,L_t)&s_{15j}(1-t_{15j})&s_{16j}(1-t_{16j})&s_{17j}(1-t_{17j})\end{array} \right].
\]  
   
   
 
 Projection function 
 The R function  Project.pop  below projects the tick population over  tmax  months. The function returns the monthly values of the size of each stage, the size of the questing population, the size of the fed stages (feeding on small hosts and on large hosts), the size of the emerging population (molting). It also returns the monthly values of the transition matrix, the survival/transition matrix  \(\mathbf{U}\) , and the fertility matrix  \(\mathbf{F}\) . 
  Project.pop <- function(tmax=3000, devframe=develop, survframe=survival, eggs_pf=1500, N0=Init, smallhost.pref=c(.9, .5, 0), needed.spots.per.tick.stage = c(1, 3, 10), maxprob.small = c(.7, .9, 0), maxprob.large=c(.3, .6, .9), small.host.capacity = SHCapacity, number.per.host.large = LHFeedingSpots, SpringFallThreshold = 7, start.month=1, HostFrame=Hosts){
  #=====================
  #ORGANIZE PARAMETERS
  #=====================
  stages <- c("EGG", "EGP", 
            "LU",  "LUP", "LFS", "LFF", "LFP",
            "NU",  "NUP", "NFS", "NFF", "NFP",
            "AU",  "AUP", "AFS", "AFF", "AFP")
  unfed.stages <- c("LU", "LUP", "NU", "NUP", "AU", "AUP")
  k <- length(stages)
  #----------------------
  #FERTILITY (Not density dependent)
  #----------------------
  #Array for F-matrices by month
  FmatArray <- array(data = 0, dim=c(12, k, k)) 
  dimnames(FmatArray)[[2]] <-  dimnames(FmatArray)[[3]] <- stages
  for(i in 1:11){
    FmatArray[i,"EGG","AFS"] <- 0.5*eggs_pf*survframe[i,"AFS"]*devframe[i,"AFS"]
    FmatArray[i,"EGG","AFF"] <- 0.5*eggs_pf*survframe[i,"AFF"]*devframe[i,"AFF"]
    FmatArray[i,"EGG","AFP"] <- 0.5*eggs_pf*survframe[i,"AFP"]*devframe[i,"AFP"]
  }
  for(i in 12){
    FmatArray[i,"EGP","AFS"] <- 0.5*eggs_pf*survframe[i,"AFS"]*devframe[i,"AFS"]
    FmatArray[i,"EGP","AFF"] <- 0.5*eggs_pf*survframe[i,"AFF"]*devframe[i,"AFF"]
    FmatArray[i,"EGP","AFP"] <- 0.5*eggs_pf*survframe[i,"AFP"]*devframe[i,"AFP"]
  }
  #----------------------
  #Organize feeding parameters for 6 unfed stages 
  #----------------------
  #LU, LUP, NU, NUP, AU, AUP
  SH.pref <- c(rep(smallhost.pref[1], 2), rep(smallhost.pref[2], 2), 
               rep(smallhost.pref[3], 2)) #Small host use
  spt <- c(rep(needed.spots.per.tick.stage[1], 2), rep(needed.spots.per.tick.stage[2], 2), 
           rep(needed.spots.per.tick.stage[3], 2))  #Spots per tick needed on small host 
  maxP.small <- c(rep(maxprob.small[1], 2), rep(maxprob.small[2], 2), 
                  rep(maxprob.small[3], 2))  #Max probability of finding small host
  maxP.large <- c(rep(maxprob.large[1], 2), rep(maxprob.large[2], 2),
                  rep(maxprob.large[3], 2))  #Max probability of finding large host
  sph.large <- c(rep(number.per.host.large[1],2), rep(number.per.host.large[2],2), rep(number.per.host.large[3],2)) #Number of ticks that can feed on each large host per month
  #----------------------
  #INITIALIZE
  #----------------------
  Nmat <- matrix(NA, ncol=tmax+1, nrow=k) #To store population size each month
  rownames(Nmat) <- stages
  Nmat[,1] <- N0 
  QuestMat <- matrix(NA, ncol=tmax, nrow=6) #To store questing population
  TMatArray <- array(NA, c(tmax, k, k)) #Store transition matrices (density dependent)
  UMatArray <- array(NA, c(tmax, k, k)) #Store survival/transition matrices (density dependent)
  dimnames(TMatArray)[[2]] <-  dimnames(TMatArray)[[3]] <- stages
  dimnames(UMatArray)[[2]] <-  dimnames(UMatArray)[[3]] <- stages
  FedMatS  <- matrix(NA, ncol=tmax, nrow=6) #store numbers feeding on small hosts 
  FedMatL  <- matrix(NA, ncol=tmax, nrow=6) #store numbers feeding on large hosts 
  rownames(QuestMat) <- rownames(FedMatL) <- rownames(FedMatS) <- unfed.stages
  month <- start.month-1 
  #=====================
  #PROJECTION LOOP 
  #=====================
  for(i in 1:tmax){#Define month within year
    month <- month+1
    if(month > 12){
      month <- 1
    }
    #----------------------
    #TRANSITIONS 
    #----------------------
    TMat <- diag(1, k, k)  
    colnames(TMat) <- rownames(TMat) <- stages
    #---------------------
    #Feeding transitions (Density dependent)
    #---------------------
    #Number of unfed ticks per stage
    Unfed <- Nmat[unfed.stages, i]
    #Questing probabilities per stage
    qprob <- devframe[month, unfed.stages]
    #~~~~~~~~~~
    #SMALL HOSTS
    #~~~~~~~~~~
    #Host availability
    HS <- HostFrame$SmallHosts[month] 
    #Number of ticks questing and finding a small host:
    nfind.small <- Unfed * qprob * SH.pref * p.find.host.small(HS, max.prob = maxP.small)
    #Available feeding spots
    feeding.spots.small <- small.host.capacity * HS 
    #Requested feeding spots 
    nreq.small <- nfind.small * spt
    #Number of fed ticks on small
    smallhostfed <- {#Sucessful feeding
      if(sum(nreq.small) > feeding.spots.small){#if not enough spots
        (nreq.small / sum(nreq.small)) * (feeding.spots.small / spt)
        } else {#if enough spots
        nreq.small / spt
        } 
      }
    #~~~~~~~~~~
    #LARGE HOSTS
    #~~~~~~~~~~
    #Host availability 
    HL <- HostFrame$LargeHosts[month] 
    #Number of ticks questing and finding a large host:
    nfind.large <- Unfed * qprob * (1 - SH.pref) * p.find.host.large(HL, max.prob = maxP.large)
    #Available feeding spots per stage
    feeding.spots.large <- sph.large*HL
    largehostfed <- smallhostfed #initiate vector
    for(j in 1:length(nfind.large)){
    if(nfind.large[j] > (feeding.spots.large)[j]){#not enough spots
      largehostfed[j] <- (feeding.spots.large/sum(sph.large))[j]
      }
    if(nfind.large[j] <= (feeding.spots.large)[j]){#enough spots
      largehostfed[j] <- nfind.large[j]
      }
    }
    #Total numbers of fed ticks
    fedticks <- largehostfed + smallhostfed #(before survival)
    FedMatS[, i] <- t(smallhostfed) #store fed on small (before survival)
    FedMatL[, i] <- t(largehostfed) #store fed on large (before survival)
    QuestMat[, i] <- t(Unfed*qprob) #store questing (before survival)
    tprob <- ifelse(Unfed > 0, fedticks/Unfed, 0)
  #----------------------------------
  #Hatching / molting probabilities
  #----------------------------------
  TMat["LU", "EGG"]  <-  devframe[month, "EGG"]   
  TMat["EGG", "EGG"] <-  1 - devframe[month, "EGG"]  
  TMat["LU", "EGP"]  <-  devframe[month, "EGP"] 
  TMat["EGP", "EGP"] <-  1 - devframe[month, "EGP"]  
  TMat["NU", "LFP"]  <-  devframe[month, "LFP"]  
  TMat["LFP", "LFP"] <-  1 - devframe[month, "LFP"] 
  TMat["NU", "LFS"]  <-  devframe[month, "LFS"]  
  TMat["LFS", "LFS"] <-  1 - devframe[month, "LFS"] 
  TMat["NU", "LFF"]  <-  devframe[month, "LFF"]  
  TMat["LFF", "LFF"] <-  1 - devframe[month, "LFF"] 
  TMat["AU", "NFP"]  <-  devframe[month, "NFP"]   
  TMat["NFP", "NFP"] <-  1 - devframe[month, "NFP"]  
  TMat["AU", "NFS"]  <-  devframe[month, "NFS"]   
  TMat["NFS", "NFS"] <-  1 - devframe[month, "NFS"] 
  TMat["AU", "NFF"]  <-  devframe[month, "NFF"] 
  TMat["NFF", "NFF"] <-  1 - devframe[month, "NFF"] 
  TMat["AFS", "AFS"] <-  1 - devframe[month, "AFS"]#Not reproducing
  TMat["AFF", "AFF"] <-  1 - devframe[month, "AFF"]#Not reproducing
  TMat["AFP", "AFP"] <-  1 - devframe[month, "AFP"]#Not reproducing
  if(month < SpringFallThreshold){#FEEDING
    TMat["LFS", "LU"]  <-  tprob$LU 
    TMat["LU", "LU"]   <-  1 - tprob$LU 
    TMat["LFS", "LUP"] <-  tprob$LUP
    TMat["LUP", "LUP"] <-  1 - tprob$LUP 
    TMat["NFS", "NU"]  <-  tprob$NU 
    TMat["NU", "NU"]   <-  1 -  tprob$NU
    TMat["NFS", "NUP"] <-  tprob$NUP
    TMat["NUP", "NUP"] <-  1 -  tprob$NUP
    TMat["AFS", "AU"]  <-  tprob$AU    
    TMat["AU", "AU"]   <-  1 - tprob$AU  
    TMat["AFS", "AUP"] <-  tprob$AUP    
    TMat["AUP", "AUP"] <-  1 - tprob$AUP  
    }
  if(month >= SpringFallThreshold){
    TMat["LFF", "LU"]  <-  tprob$LU 
    TMat["LU", "LU"]   <-  1 - tprob$LU 
    TMat["LFF", "LUP"] <-  tprob$LUP  
    TMat["LUP", "LUP"] <-  1 - tprob$LUP  
    TMat["NFF", "NU"]  <-  tprob$NU   
    TMat["NU", "NU"]   <-  1 - tprob$NU  
    TMat["NFF", "NUP"] <-  tprob$NUP  
    TMat["NUP", "NUP"] <-  1 - tprob$NUP  
    TMat["AFF", "AU"]  <-  tprob$AU 
    TMat["AU", "AU"]   <-  1 - tprob$AUP
    TMat["AFF", "AUP"] <-  tprob$AUP
    TMat["AUP", "AUP"] <-  1 - tprob$AUP 
    }
if(month==12){#Transitions Dec/Jan including shifts to "previous"
  TMat <- diag(0, k, k) 
  colnames(TMat) <- rownames(TMat) <- stages
  TMat["EGP", "EGG"] <- 1 -  devframe[month, "EGG"]    
  TMat["LUP", "EGG"] <- devframe[month,"EGG"]
  TMat["EGP", "EGP"] <- 1 - devframe[month,"EGP"]
  TMat["LUP", "EGP"] <- devframe[month,"EGP"]
  TMat["LUP", "LU"] <-  1 - tprob$LU
  TMat["LFP", "LU"] <-  tprob$LU
  TMat["LUP", "LUP"] <- 1 - tprob$LUP
  TMat["LFP", "LUP"] <- tprob$LUP
  TMat["LFP", "LFF"] <-  1 - devframe[month,"LFF"]
  TMat["NUP", "LFF"] <-  devframe[month,"LFF"]
  TMat["LFP", "LFS"] <-  1 - devframe[month,"LFS"]
  TMat["NUP", "LFS"] <-  devframe[month,"LFS"]
  TMat["LFP", "LFP"] <- 1 - devframe[month,"LFP"]
  TMat["NUP", "LFP"] <- devframe[month,"LFP"]
  TMat["NUP", "NU"] <-  1 - tprob$NU
  TMat["NFP", "NU"] <-  tprob$NU
  TMat["NUP", "NUP"] <- 1-tprob$NUP
  TMat["NFP", "NUP"] <- tprob$NUP
  TMat["NFP", "NFF"] <-  1 - devframe[month,"NFF"]
  TMat["AUP", "NFF"] <-  devframe[month,"NFF"]
  TMat["NFP", "NFS"] <-  1 - devframe[month,"NFS"]
  TMat["AUP", "NFS"] <-  devframe[month,"NFS"]
  TMat["NFP", "NFP"] <- 1-devframe[month,"NFP"]
  TMat["AUP", "NFP"] <- devframe[month,"NFP"]
  TMat["AUP", "AU"] <-  1 - tprob$AU
  TMat["AFP", "AU"] <-  tprob$AU
  TMat["AUP", "AUP"] <- 1-tprob$AUP
  TMat["AFP", "AUP"] <- tprob$AUP 
  TMat["AFP", "AFS"] <-  1 - devframe[month, "AFS"]
  TMat["AFP", "AFF"] <-  1 - devframe[month, "AFF"]
  TMat[ "AFP", "AFP"] <-  1 - devframe[month, "AFP"]
  }
  TMatArray[i,,] <- TMat #Store current transition matrix
  #PROJECTION MATRIX
    UMat <- TMat * as.numeric(t(matrix(survframe[month, 3:(k+2)], ncol=k, nrow=k)))
    UMatArray[i,,] <- UMat #Store current survival/transition matrix
    FMat <- FmatArray[month,,] 
    AMat <- UMat + FMat 
    Nmat[,i+1] <- Nmat[,i]%*%t(AMat) #Project population to next month
  }#end loop over months
  namemonths <- c(month.abb[start.month:12],rep(month.abb,tmax+1))[1:(tmax+1)]
  TotalPop <- data.frame(Nmat)
  Questing <- data.frame(QuestMat)
  FedOnSmall <- data.frame(FedMatS)
  FedOnLarge <- data.frame(FedMatL)
  names(TotalPop) <- namemonths   
  names(Questing) <-   names(FedOnSmall) <-   names(FedOnLarge) <-   namemonths[1:tmax]
  dimnames(TMatArray)[[1]] <- namemonths[1:tmax]
  dimnames(UMatArray)[[1]] <- namemonths[1:tmax]
  return(list("Pop" = TotalPop, 
              "QuestingPop" = Questing, 
              "FedOnSmallHost" = FedOnSmall, 
              "FedOnLargeHost" = FedOnLarge,
              "FMatArray" = FmatArray,
              "UMats" = UMatArray,
              "TMats" = TMatArray))
  }  
   
 
 
 
 
 
 S3 Analyses 
 
 S3.1 Baseline model 
 The following code projects the baseline model over 3000 months: 
  SHLevel <- 10000 #Starting value in January, small host adults
LHLevel <- 500 #Large host level
SHCapacity <- 500 #Capacity of small hosts to feed  larvae and nymphs per month
LHFeedingSpots <- c(5000, 5000, 5000) #Number of larvae, nymphs and adults that can feed on each large host per month

Hosts <- data.frame(Month = month.abb)
Hosts$LargeHosts <- rep(1,12)*LHLevel
Hosts$SmallHosts <- popsize.SH(N0=SHLevel, VR=vitalrates.SH)$Adults
Hosts$Month <- factor(Hosts$Month,levels=month.abb)

run <- Project.pop(tmax=3000, devframe=develop, survframe=survival, eggs_pf=1500, N0=Init, smallhost.pref=c(.9, .5, 0), needed.spots.per.tick.stage = c(1, 3, 10), maxprob.small = c(.7, .9, 0), maxprob.large=c(.3, .6, .9), small.host.capacity = SHCapacity, number.per.host.large = LHFeedingSpots, SpringFallThreshold = 7, start.month=1, HostFrame=Hosts)  
   
 
 S3.1.1 Projected population results 
 
 Stage sizes in August 
 The plot below shows how the projection approaches a stable seasonal cycle with the same size each year for month (August is chosen for this example). With the initial values used this takes around 50 years. 
   
 
 
Figure S6: August population for year 1 to 200, for the baseline model (initial stage size 10000 in each overwintering stage and 0 in the other stages).
 
 
   
 
 
 Stage sizes per month 
 Extract the monthly stage size after 198, 199 and 200 years (to verify these are the same and a stable seasonal cycle is reached). 
   
 
 
Figure S7: Size of each stage against month in the baseline model after 200 years (after a stable seasonal cycle is reached). The vertical grey lines indicate the threshold month for spring and fall feeding (July).
 
 
   
 
 
 Monthly questing population 
 Extract the number of questing larvae, nymphs and adults per month, in year 200 (after the stable cycle is reached). 
   
   
 
 
Figure S8: Questing population of each stage per month, in the baseline model after 200 years (after a stable seasonal cycle is reached). The vertical grey lines indicate the threshold month for spring and fall feeding (July).
 
 
   
 
 
 Feeding individuals per month 
 Extract the number of questing larvae, nymphs and adults that feed in a given month, for year 200 (after the stable cycle is reached). These numbers can be compared to the questing population for evaluation of whether the number feeding results mostly from the number questing or from the host availability. 
   
   
 
 
Figure S9: Numbers feeding on each host type (small or large) per month, in the baseline model after 200 years (after a stable seasonal cycle is reached). The vertical grey lines indicate the threshold month for spring and fall feeding (July).
 
 
   
 
 
 Host capacity used 
 The code below extracts the proportion of the total available feeding spots are used per month at year 200 (after stable cycle has been reached), by all ticks irrespective of stage. A proportion of 1 means the ticks were competing for space on the hosts. 
   
 
 
Figure S10: The proportion of available host capacity used per month, for small and large hosts after 200 years (after a stable seasonal cycle is reached). Calculated as number of used feeding spots divided by number of available feeding spots per host type per month.
 
 
   
 We can also consider what proportion of the total available feeding spots are used per tick stage: 
   
 
 
Figure S11: The proportion of available host capacity used per stage, for small and large hosts, after 200 years (after a stable seasonal cycle is reached). The sum of the stage-specific proportions equals the total proportion of the host capacity used.
 
 
   
 Figure S11 shows that in months of high competition for the small host space (June, July) larvae use most of the available spots. 
 The following code calculates the proportion of available feeding spots to each tick stage that was actually used by each stage: 
   
 
 
Figure S12: The proportion of available host capacity for each stage that is used per stage, for small and large hosts, after 200 years (after a stable seasonal cycle is reached). Calculated for each host type as the number of used feeding spots per stage divided by number of available feeding spots per stage per month.
 
 
   
 
 
 Fed population 
 The code below extracts the number of individuals in each fed stage (LFS, LFF, LFP, NFS, NFF, NFP, AFS, AFF, AFP) per month. This should not be confused with the number of individuals that feed in a given month (Fig. S9). Individuals enter a fed stage by feeding, and remain until they leave it by death, moulting, or transition to an overwintering stage. By tracking the fed stages we get an overview of how many ticks are in the spring fed stages compared to fall fed or overwintered stages. 
   
 
 
Figure S13: Size of the spring and fall fed population (larvae, nymphs and adults) in years 198 to 200.
 
 
   
 
 
 Direct and delayed development 
 In the new generation of unfed individuals that appear each year (stages EGG, LU, NU, and AU), we can ask how how many are produced from individuals that fed last fall, or hatch from eggs that were laid last fall (previous, representing delayed development), versus from individuals feeding or eggs laid in the current year (representing direct development). 
   
 
 
Figure S14: Eggs laid from adults fed current year or previous year, and newly emerged individuals developed from individuals feeding that fed in the current or previous year, after 200 years (after a stable seasonal cycle is reached). The vertical grey lines indicate the threshold month for spring and fall feeding (July).
 
 
   
 The plot below shows the same without the egg stage included. 
   
 
 
Figure S15: Newly emerged individuals developed from individuals feeding that fed in the current or previous year, after 200 years (after a stable seasonal cycle is reached). The vertical grey lines indicate the threshold month for spring and fall feeding (July).
 
 
   
 
 
 Combined plot 
   
 
 
Figure S16: Results from the baseline model, figure 3 in main text. A. Questing population (current/previous), B. Numbers feeding per month (on small/large host), C. Fed population from spring, fall, and from previous year, and D. Emerging unfed individuals per month (direct / delayed development). The vertical grey lines indicate the threshold month for spring and fall feeding (July).
 
 
   
 
 
 
 S3.1.2 Life history and demographic calculations 
 The code below calculates the following parameters that describe the life history and population structure when the stable cycle is reached: 
 
 Long-term population growth rate  \(\lambda\)  (should be 1) 
 Stable stage structure 
 Reproductive values 
 Mean lifespan for an average offspring 
 Variance in lifespan for an average offspring 
 Mean remaining lifespan for each stage 
 Variance in remaining lifespan for each stage 
 Mean lifetime reproductive output (LRO) for an average offspring,  \(R_0\)  (should be 1) 
 Variance in (LRO) for an average offspring 
 Mean lifetime reproductive output (LRO) for each stage 
 Variance in (LRO) for each stage 
 Generation time 
 
 These parameters are calculated for annual matrices with different annual census months (from January to December). Some parameters are the same regardless of census time, while others depend on the month of annual census. 
   
 
 Parameters not depending on stage 
   
 
  Table S3:  Life history parameters for the baseline model, calculated for the annual projection matrix (and its main component) extracted when the population has reached its stable seasonal cycle. 
 
 
 
 
 
 
 
 
 
 
 
 StartMonth 
 lambda 
 Mean_LRO 
 SD_LRO 
 Mean_Lifespan 
 SD_Lifespan 
 Gen_Time 
 
 
 
 
 January 
 1 
 1 
 17 
 0.135 
 0.400 
 3.718 
 
 
 February 
 1 
 1 
 17 
 0.138 
 0.405 
 3.718 
 
 
 March 
 1 
 1 
 16 
 0.141 
 0.410 
 3.718 
 
 
 April 
 1 
 1 
 16 
 0.144 
 0.415 
 3.718 
 
 
 May 
 1 
 1 
 15 
 0.166 
 0.440 
 3.718 
 
 
 June 
 1 
 1 
 15 
 0.201 
 0.475 
 3.718 
 
 
 July 
 1 
 1 
 13 
 0.304 
 0.556 
 3.718 
 
 
 August 
 1 
 1 
 15 
 0.278 
 0.544 
 3.718 
 
 
 September 
 1 
 1 
 18 
 0.223 
 0.505 
 3.718 
 
 
 October 
 1 
 1 
 19 
 0.174 
 0.453 
 3.718 
 
 
 November 
 1 
 1 
 18 
 0.144 
 0.413 
 3.718 
 
 
 December 
 1 
 1 
 18 
 0.132 
 0.396 
 3.718 
 
 
 
 The mean lifetime reproductive output is 1 and the growth rate lambda is 1 for all months, as they should be for matrices extracted when the stable seasonal cycle is reached in a density dependent model. 
   
 
 
 Stable stage structure 
   
 
 
Figure S17: Stable structure at equilibrium, calculated from annual projection matrices with different months as annual census time, for the baseline model.
 
 
   
 Most of the tick population is found as eggs or unfed larvae. 
   
 
 
 Reproductive values 
   
 
 
Figure S18: Reproductive values calculated from annual projection matrices starting at each month, for the baseline model.
 
 
   
 The reproductive value tell us which stage is contributing most to future population growth. It varies depending on census month, but is generally highest for fed adults and lowest for eggs and larvae. 
   
 
 
 Mean and variance of remaining lifespan 
   
 
 
Figure S19: Mean remaining lifespan (in years) by stage, calculated from annual matrices starting at each month, for the baseline model.
 
 
   
 The mean remaining lifespan is highest in the fed nymph stage. Since reproduction means death in this model, the mean remaining lifespan is low for adult classes close to reproduction. 
   
   
 
 
Figure S20: Standard deviation of remaining lifetime by stage, calculated from annual matrices starting at each month, for the baseline model.
 
 
   
 The variance in remaining lifespan is generally higher in the larvae and nymph stages. 
 
 
 Mean and variance of lifetime reproductive output per stage 
   
 
 
Figure S21: Mean remaining lifetime reproductive output by stage, calculated from annual matrices starting at each month, for the baseline model. Note that in annual matrices offspring are counted in different stages.
 
 
   
 The mean lifetime reproductive output increases the closer the stage is to a reproductive stage, and is generally highest in the adult fed stages. If a tick has reach such a stage, the expected reproductive output is very high compared to a tick in for instance the egg or larval stage, which does not have high chances of reaching the adult stage. 
   
   
 
 
Figure S22: Standard deviation of remaining lifetime reproductive output by stage, calculated from annual matrices starting at each month, for the baseline model. Note that in annual matrices offspring are counted in different stages.
 
 
   
 
 
 
 
 S3.2 Vary host parameters 
 
 S3.2.1 Vary host levels 
 In these analyses we first vary the small host population size in January (small host level), then the large host level (which is constant across months). 
   
   
 
 
Figure S23: Figure 4 in the main text. Questing population with varying small host level (A) and varying large host level (B), all other parameters are the same as in the baseline model
 
 
   
 
 
 S3.2.2 Vary small host utilization 
 In these analyses we vary the small host utilization parameter of larvae and nymphs, one by one. In the baseline model the small host utilization is 0.9 in larvae and 0.5 in nymphs. 
   
 
 
Figure S24: Figure 5 in the main text. Questing population with varying small host utilization by nymphs (A) and by larvae (B). All other parameters are the same as in the baseline model.
 
 
   
 
 
 
 S3.3 Constant small host 
 Here we use the same parameters as in the baseline scenario, except that the small host availability is constant across months (equal to the mean availability of the baseline scenario). Some results are plotted with the results from the baseline scenario (S3) as comparison. 
  HostsConstant <- data.frame(Month = month.abb)
HostsConstant$LargeHosts <- rep(1,12)*LHLevel
HostsConstant$SmallHosts <- popsize.SH(N0=SHLevel*.9769*2, VR=vitalrates.SH.Const, Constant=TRUE)$Adults
HostsConstant$Month <- factor(HostsConstant$Month,levels=month.abb)

run2 <- Project.pop(tmax=3000, devframe=develop, survframe=survival, eggs_pf=1500, N0=Init, smallhost.pref=c(.9, .5, 0), needed.spots.per.tick.stage = c(1, 3, 10), maxprob.small = c(.7, .9, 0), maxprob.large=c(.3, .6, .9), small.host.capacity = SHCapacity, number.per.host.large = LHFeedingSpots, SpringFallThreshold = 7, start.month=1, HostFrame=HostsConstant)  
   
 
 S3.3.1 Projected population results 
 
 Stage sizes in August 
   
 
 
Figure S25: Equilibrium population size, in the model with constant small host availability compared to the baseline model with seasonal small host availability.
 
 
   
 
 
 Stage sizes per month 
   
 
 
Figure S26: Size of each stage against month at equilibrium, in the model with constant small host availability compared to the baseline model with seasonal small host availability. The vertical grey lines indicate the threshold month for spring and fall feeding (July).
 
 
   
 
 
 Questing population 
   
 
 
Figure S27: Questing population of each stage per month at equilibrium, in the model with constant small host availability compared to the baseline model with seasonal small host availability. The vertical grey lines indicate the threshold month for spring and fall feeding (July).
 
 
   
 
 
 Feeding individuals per month 
   
 
 
Figure S28: Size of the fed population (larvae, nymphs and adults) on each host type (small or large) per month at equilibrium, in the model with constant small host availability compared to the baseline model with seasonal small host availability. The vertical grey line indicates the threshold month for spring and fall feeding (July).
 
 
   
 
 
 Host capacity use 
 Available host capacity used: 
   
 
 
Figure S29: The proportion of available host capacity used per month, for small and large hosts, compared to the scenario with seasonal small host. Calculated as number of used feeding spots divided by number of available feeding spots per host type per month.
 
 
   
 Available host capacity used per stage: 
   
 
 
Figure S30: The proportion of available host capacity used per stage, for small and large hosts, compared to the scenario with seasonal small host. The sum of the stage-specific proportions equals the total proportion of the host capacity used.
 
 
   
 Available host capacity to each stage that is used by the stage: 
   
 
 
Figure S31: The proportion of available host capacity for each stage that is used per stage, for small and large hosts, compared to the scenario with seasonal small host. Calculated for each host type as the number of used feeding spots per stage divided by number of available feeding spots per stage per month.
 
 
   
 
 
 Fed population 
   
 
 
Figure S32: Size of the spring and fall fed population (larvae, nymphs and adults) at equilibrium, in the model with constant small host availability compared to the baseline model with seasonal small host availability. The vertical grey line indicates the threshold month for spring and fall feeding (July).
 
 
   
 
 
 Direct and delayed development 
   
 
 
Figure S33: Eggs laid from adults fed current year or previous year, and newly emerged individuals developed from individuals feeding that fed in the current or previous year, at equilibrium in the model with constant small host availability. The vertical grey line indicates the threshold month for spring and fall feeding (July).
 
 
   
   
 
 
Figure S34: Newly emerged individuals developed from individuals feeding that fed in the current or previous year at equilibrium, in the model with constant small host availability compared to the baseline model with seasonal small host availability. The vertical grey line indicates the threshold month for spring and fall feeding (July).
 
 
   
 
 
 Combined plot 
   
 
 
Figure S35: Equilibrium results in the model with constant small host availability compared to the baseline model with seasonal small host availability. A. Questing population (current/previous), B. Numbers feeding per month (on small/large host), C. Fed population from spring, fall, and from previous year, and D. Emerging unfed individuals per month (direct / delayed development). The vertical grey lines indicate the threshold month for spring and fall feeding (July).
 
 
   
 
 
 
 S3.3.2 Life history and demographic calculations 
   
 
  Table S4:  Life history parameters for the model with constant small host availability, calculated for the annual projection matrix (and its main component) extracted when the population has reached its stable seasonal cycle. 
 
 
 
 
 
 
 
 
 
 
 
 StartMonth 
 lambda 
 Mean_LRO 
 SD_LRO 
 Mean_Lifespan 
 SD_Lifespan 
 Gen_Time 
 
 
 
 
 January 
 1 
 1 
 17 
 0.126 
 0.383 
 3.612 
 
 
 February 
 1 
 1 
 17 
 0.128 
 0.387 
 3.612 
 
 
 March 
 1 
 1 
 16 
 0.131 
 0.392 
 3.612 
 
 
 April 
 1 
 1 
 16 
 0.134 
 0.396 
 3.612 
 
 
 May 
 1 
 1 
 15 
 0.156 
 0.422 
 3.612 
 
 
 June 
 1 
 1 
 15 
 0.191 
 0.459 
 3.612 
 
 
 July 
 1 
 1 
 13 
 0.297 
 0.542 
 3.612 
 
 
 August 
 1 
 1 
 15 
 0.275 
 0.533 
 3.612 
 
 
 September 
 1 
 1 
 18 
 0.220 
 0.495 
 3.612 
 
 
 October 
 1 
 1 
 19 
 0.168 
 0.441 
 3.612 
 
 
 November 
 1 
 1 
 18 
 0.137 
 0.399 
 3.612 
 
 
 December 
 1 
 1 
 18 
 0.124 
 0.379 
 3.612 
 
 
 
   
   
 
 
 
 S3.4 Southern scenario 
 In this scenario we consider a southern life history assumed to represent the southern part of the distribution range of  Ixodes ricinus  in Europe The parameters are chosen to fit approximately the questing phenology reported by Dantas-Torres et al. 2013 for a population from southern Italy, and by Estrada-Peña et al. (2004) for a population from north-central Spain. We assume lower survival than in the baseline northern scenario, due to increased risk of desiccation as described by Estrada-Peña & Estrada-Sanchez (2014). We do not know the exact probabilities for questing, molting and reproduction, but we assume that the life cycle progression is overall faster than in northern poplations due to higher mean temperature in all months. The overall level of these transition rates were thus tuned in initial model development to obtain 1) a viable population over time (approaching a stable seasonal cycle), 2) a generation time of approximately 2 years, and 3) that the population size of questing larvae is an order of magnitude higher than that of questing nymphs. 
 For this scenario we assume that all larvae quest in the first year when they are hatched (no overwintering of unfed larvae). The spring/fall treshold is set to August, but does not affect survival of spring fed individuals (as in the Northern model) and the likelihood of molting is also set to be the same. In other words, adjusting the spring / fall threshold has no influence on this population. 
   
 
 S3.4.1 Tick parameters 
 
 Survival and transitions 
   
   
   
 
 
Figure S36: Baseline values of survival and density independent transition probabilities in the different stages, for the Southern life history. All individuals die after reproducing so in reproductive stages (‘AFS’, ‘AFF’, ‘AFP’) the transition probability corresponds to the probability of reproducing and dying. Seasonal density independent baseline probabilities of hatching (eggs), questing (unfed stages), molting (fed stages of larvae and nymphs) and reproducing / dying (adult fed stages). Feeding is a density dependent process depending on host availability in addition to questing probability (specified below).
 
 
   
   
 
 
Figure S37: Comparison of the density independent transition probabilities in the baseline Northern and the alternative Southern life history.
 
 
   
 
 
 Egg number per female 
 We assume the same fecundity, 1500 eggs per female, as in the baseline scenario. 
   
 
 
 
 S3.4.2 Projection results 
  HostsSouthern <- data.frame(Month = month.abb)
HostsSouthern$LargeHosts <- rep(1,12)*LHLevel
HostsSouthern$SmallHosts <-  popsize.SH(N0=SHLevel*.55,VR=vitalrates.SH.Southern)$Adults
HostsSouthern$Month <- factor(Hosts$Month,levels=month.abb)

run.southern <- Project.pop(tmax=3000, devframe=develop.southern, survframe=survival.southern, eggs_pf=1500, N0=Init, smallhost.pref=c(.9, .5, 0), needed.spots.per.tick.stage = c(1, 3, 10), maxprob.small = c(.7, .9, 0), maxprob.large=c(.3, .6, .9), small.host.capacity = SHCapacity, number.per.host.large = LHFeedingSpots, SpringFallThreshold = 8, start.month=1, HostFrame = HostsSouthern)  
   
 
 Stage sizes in August 
   
 
 
Figure S38: August size in the southern scenario, compared to the baseline northern model.
 
 
   
 
 
 Stage sizes per month 
   
 
 
Figure S39: Monthly stage size in the southern scenario, compared to the baseline northern model. The vertical grey lines indicate the threshold month for spring and fall feeding (July for northern, August for southern).
 
 
   
 
 
 Questing population 
   
 
 
Figure S40: Questing population of each stage per month, comparing the northern baseline model to the alternative southern scenario. The vertical grey lines indicate the threshold month for spring and fall feeding (July for Northern, August for Southern).
 
 
   
 
 
 Feeding population per month 
   
 
 
Figure S41: Size of the fed population (larvae, nymphs and adults) on each host type (small or large) per month, comparing the northern baseline model with the southern scenario. The vertical grey lines indicate the threshold month for spring and fall feeding (July for Northern, August for Southern).
 
 
   
 
 
 Host capacity use 
 Available host capacity used: 
   
 
 
Figure S42: The proportion of available host capacity used per month, for small and large hosts, compared to the baseline northern scenario. Calculated as number of used feeding spots divided by number of available feeding spots per host type per month.
 
 
   
 Available host capacity used per stage: 
   
 
 
Figure S43: The proportion of available host capacity used per stage, for small and large hosts, compared to the baseline northern scenario. The sum of the stage-specific proportions equals the total proportion of the host capacity used.
 
 
   
 Available host capacity to each stage that is used by the stage: 
   
 
 
Figure S44: The proportion of available host capacity for each stage that is used per stage, for small and large hosts, compared to the baseline northern scenario. Calculated for each host type as the number of used feeding spots per stage divided by number of available feeding spots per stage per month.
 
 
   
 
 
 Fed population 
   
 
 
Figure S45: Size of the spring and fall fed population (larvae, nymphs and adults), comparing the northern baseline model to the alternative southern scenario. The vertical grey lines indicate the threshold month for spring and fall feeding (July for Northern, August for Southern).
 
 
   
 
 
 Direct and delayed development 
   
 
 
Figure S46: Eggs laid from adults fed current year or previous year, and newly emerged individuals developed from individuals feeding that fed in the current or previous year, comparing the northern baseline model to the alternative southern scenario. . The vertical grey lines indicate the threshold month for spring and fall feeding (July for Northern, August for Southern).
 
 
   
 
 
Figure S47: Newly emerged individuals developed from individuals feeding that fed in the current or previous year, comparing the northern baseline model to the alternative southern scenario. The vertical grey lines indicate the threshold month for spring and fall feeding (July for Northern, August for Southern).
 
 
   
 
 
 Combined plot 
   
 
 
Figure S48: Equilibrium results for the Northern baseline model with small host seasonality compared to the alternative Southern scenario with constant small host. A. Questing population (current/previous), B. Numbers feeding per month (on small/large host), C. Fed population from spring, fall, and from previous year, and D. Emerging unfed individuals per month (direct / delayed development). The vertical grey lines indicate the threshold month for spring and fall feeding (July for Northern, August for Southern).
 
 
   
   
 
 
Figure S49: Equilibrium results for the southern scenario alone. A. Questing population (current/previous), B. Numbers feeding per month (on small/large host), C. Fed population from spring, fall, and from previous year, and D. Emerging unfed individuals per month (direct / delayed development). The vertical grey lines indicate the threshold month for spring and fall feeding (August).
 
 
   
 
 
 
 S3.4.3 Life history and demographic calculations 
 
  Table S5:  Life history parameters for the model with constant small host availability, calculated for the annual projection matrix (and its main component) extracted when the population has reached its stable seasonal cycle. 
 
 
 
 
 
 
 
 
 
 
 
 StartMonth 
 lambda 
 Mean_LRO 
 SD_LRO 
 Mean_Lifespan 
 SD_Lifespan 
 Gen_Time 
 
 
 
 
 January 
 1 
 1 
 7 
 0.052 
 0.243 
 2.23 
 
 
 February 
 1 
 1 
 7 
 0.056 
 0.251 
 2.23 
 
 
 March 
 1 
 1 
 7 
 0.058 
 0.257 
 2.23 
 
 
 April 
 1 
 1 
 7 
 0.075 
 0.289 
 2.23 
 
 
 May 
 1 
 1 
 8 
 0.078 
 0.293 
 2.23 
 
 
 June 
 1 
 1 
 10 
 0.061 
 0.261 
 2.23 
 
 
 July 
 1 
 1 
 10 
 0.058 
 0.250 
 2.23 
 
 
 August 
 1 
 1 
 10 
 0.056 
 0.241 
 2.23 
 
 
 September 
 1 
 1 
 10 
 0.049 
 0.227 
 2.23 
 
 
 October 
 1 
 1 
 10 
 0.037 
 0.201 
 2.23 
 
 
 November 
 1 
 1 
 10 
 0.030 
 0.184 
 2.23 
 
 
 December 
 1 
 1 
 10 
 0.029 
 0.180 
 2.23 
 
 
 
   
   
 
 
 
 
 References 
 Andreassen, H.P., Sundell, J., Ecke, F., Halle, S., Haapakoski, M., Henttonen, H., Huitu, O., Jacob, J., Johnsen, K., Koskela, E., Luque-Larena, J.J., Lecomte, N., Leirs, H., Mariën, J., Neby, M., Rätti, O., Sievert, T., Singleton, G.R., van Cann, J., Vanden Broecke, B., Ylönen, H., 2021. Population cycles and outbreaks of small rodents: Ten essential questions we still need to solve. Oecologia 195, 601–622.  https://doi.org/10.1007/s00442-020-04810-w  
 Crespin, L., R. Verhagen, N. Chr. Stenseth, N. G. Yoccoz, A.-C. Prévot-Julliard, and J.-D. Lebreton. 2002. Survival in fluctuating bank vole populations: seasonal and yearly variations. Oikos 98:467–479.  https://doi.org/10.1034/j.1600-0706.2002.980311.x  
 Dantas-Torres, F., Otranto, D., 2013. Seasonal dynamics of  Ixodes ricinus  on ground level and higher vegetation in a preserved wooded area in southern Europe. Vet. Parasitol. 192, 253–258.  https://doi.org/10.1016/j.vetpar.2012.09.034  
 Dautel, H., and W. Knülle. 2010. Embryonic diapause and cold hardiness of  Ixodes ricinus  eggs (Acari: Ixodidae). Pages 327–331 in M. W. Sabelis and J. Bruin, editors. Trends in Acarology. Springer Netherlands, Dordrecht.  https://doi.org/10.1007/978-90-481-9837-5_52  
 Estrada-Peña, A., J. M. Martinez, C. Sanchez Acedo, J. Quilez, and E. Del Cacho. 2004. Phenology of the tick,  Ixodes ricinus , in its southern distribution range (central Spain). Medical and Veterinary Entomology 18:387–397. 
 Estrada-Peña, A., Estrada-Sánchez, D., 2014. Deconstructing  Ixodes ricinus : a partial matrix model allowing mapping of tick development, mortality and activity rates. Med. Vet. Entomol. 28, 35–49.  https://doi.org/10.1111/mve.12009  
 Gray, J.S., 1981. The fecundity of  Ixodes ricinus  (L.) (Acarina: Ixodidae) and the mortality of its developmental stages under field conditions. Bull. Entomol. Res. 71, 533–542.  https://doi.org/10.1017/S0007485300008543  
 Gray, J.S., Kahl, O., Lane, R.S., Levin, M.L., Tsao, J. I. 2016. Diapause in ticks of the medically important  Ixodes ricinus  species complex. Ticks. Tick. Borne. Dis. 7:992-1003.  http://dx.doi.org/10.1016/j.ttbdis.2016.05.006  
 Grigoryeva, L.A., Shatrov, A.B., 2022. Life cycle of the tick  Ixodes ricinus  (L.) (Acari: Ixodidae) in the north-west of Russia. Syst. Appl. Acarol. 27, 538–550.  https://doi.org/10.11158/saa.27.3.11  
 Hernandez, C. M., Ellner, S. P., Snyder, R. E., & Hooker, G. (2024). Supplemental code and data for Hernandez et al. “The natural history of luck: A synthesis study of structured population models”.  https://doi.org/10.5281/zenodo.10527715  
 Innes, D. G. l., and J. S. Millar. 1994. Life histories of  Clethrionomys  and  Microtus  (Microtinae). Mammal Review 24:179–207.  https://doi.org/10.1111/j.1365-2907.1994.tb00142.x  
 Nyholm, N. E. I., and P. Meurling. 1979. Reproduction of the bank vole,  Clethrionomys glareolus , in Northern and Southern Sweden during several seasons and in different phases of the vole population cycle. Holarctic Ecology 2:12–20. 
 Kahl, O., Gray, J. S. 2023. The biology of  Ixodes ricinus  with emphasis on its ecology. Ticks. Tick. Borne. Dis. 14:102114.
 https://doi.org/10.1016/j.ttbdis.2022.102114  
 Randolph, S. E., 2004. Tick ecology : processes and patterns behind the epidemiological risk posed by ixodid ticks as vectors. Parasitology 129:S37-S65.  https://doi.org/10.1017/S0031182004004925  
 


 
 

 

 

 

 

 

 

 
 

 
 
